# Decoding estrogen receptor and GPER biology: structural insights and therapeutic advances in ERα−positive breast cancer

**DOI:** 10.3389/fonc.2025.1513225

**Published:** 2025-06-26

**Authors:** Taniya Saha, Kiven Erique Lukong

**Affiliations:** Department of Biochemistry, Microbiology, and Immunology, University of Saskatchewan, Saskatoon, SK, Canada

**Keywords:** breast cancer, estrogen signaling, ERα, GPER, endocrine resistance, SERM, SERD

## Abstract

Classical estrogen receptors, ERα and ERβ, along with the membrane-bound G-protein-coupled estrogen receptor (GPER), play critical roles in driving ERα−positive breast cancer (BC). Clinical management of this subtype relies on endocrine therapy (ET), which targets ER signaling through selective estrogen receptors modulators (SERMs), degraders (SERDs), and aromatase inhibitors (AIs). While ET has significantly reduced recurrence and mortality rates, acquired resistance remains a major therapeutic challenge. Activating *ESR1* mutations, which encode constitutively active ERα variants, are detected in 30-50% of therapy-resistant metastatic ERα−positive BC and serve as emerging biomarkers of poor prognosis. These hot-spot mutations stabilize ERα in its agonist conformation, thereby enabling ligand-independent transcriptional activation. Understanding the conformational constraints that keep wild-type ERα in an “off-state” in the absence of ligand—and how activating *ESR1* mutations disrupt these regulatory mechanisms—is critical for developing effective targeted therapies. Concurrently, GPER-mediated non-genomic signaling, often inadvertently activated by SERMs and SERDs, contributes to tamoxifen resistance. This review explores the structural and functional intricacies of ERα, the impact of *ESR1* mutations on its ligand-binding domain (ERα−LBD) and their contribution to ET resistance, and the role of GPER-mediated signaling in ERα−positive BC. We further highlight recent advances in next-generation therapeutics targeting both ERα mutants and GPER, which may offer a more effective, integrated strategy to overcome ET resistance.

## 1 Introduction

According to the American Cancer Society’s Breast Cancer Facts & Figures 2024–2025, an estimated 310,720 new cases of invasive breast cancer and 56,500 cases of ductal carcinoma *in situ* (DCIS) are expected to be diagnosed among U.S. women in 2024. The latest GLOBOCAN 2022 estimates from the International Agency for Research on Cancer identify breast cancer (BC) as the second most commonly diagnosed cancer worldwide—following lung cancer—and the most frequently diagnosed cancer in women, with approximately 2.3 million new cases, accounting for 11.5% of all cancer diagnoses (1–6). At the molecular level, genomic and transcriptomic profiling—based on the expression of estrogen receptors (ER), progesterone receptors (PR), and HER2—classifies breast tumors into four main subtypes: luminal A (ER+ and/or PR+, HER2−, Ki67 <14%), luminal B (ER+ and/or PR+, HER2+ or HER2−, Ki67 >14%), HER2-enriched (ER−, PR−, HER2+), and basal-like/triple-negative (ER−, PR−, HER2−) (7). Among these, luminal-A and luminal-B subtypes predominantly express ER, with approximately 70% of newly diagnosed breast cancers being ER-positive (ER+) (8, 9). In ER+ tumors, ERα serves as the principal oncogenic driver, typically requiring estrogen (E2) for activation. However, deregulated ER expression and aberrant E2-ER interactions contribute significantly to disease progression, making endocrine therapy (ET)—which works by blocking ERα activity—a mainstay treatment for this subtype. ERs are classified into two main families: (1) the classical ERs, ERα and ERβ, which are ligand-induced nuclear receptors with a high degree of amino acid homology, functioning through E2-mediated genomic signaling (10, 11); and (2) the G-protein-coupled receptor 30 (GPR30) or G protein-coupled estrogen receptor (GPER), a family of membrane receptors that mediate E2-induced rapid non-genomic signaling and function as transcription regulators via the second messenger system (12, 13). Conventionally, ET relies on six major therapeutic classes: selective estrogen receptor modulators (SERMs), selective estrogen receptor degraders (SERDs), aromatase inhibitors (AIs), CDK4/6 inhibitors, used in combination with SERDs/AIs, and mTORC1 inhibitors in combination with AIs, as discussed below (14–17).

Tamoxifen, the first SERM, is an ERα antagonist that competitively inhibits estrogen binding to ERα and was approved by the FDA in 1972 for both pre- and postmenopausal BC patients (18, 19). Orally administered tamoxifen is extensively metabolized into active forms—4-hydroxytamoxifen (4OHT) and 4-hydroxy-N-desmethyl-tamoxifen (endoxifen)—by cytochrome P450 (CYP) enzymes such as CYP3A4 and CYP2D6 (20). However, genetic polymorphisms in CYP2D6, observed in a significant number of BC patients, lead to variable tamoxifen metabolism, contributing to inconsistent therapeutic outcomes (21, 22). Notably, Z-endoxifen (ENDX), the most active isomer of endoxifen, has demonstrated promising antitumor activity and manageable toxicity compared to tamoxifen in ERα-positive metastatic breast cancer (MBC) patients harboring *ESR1* mutations—the gene encoding ERα (23). Recognition of genetic variability in tamoxifen metabolism led to the development of toremifene, a first-generation SERM that differs from tamoxifen by a single chlorine atom (24, 25). SERMs are known for their tissue-specific dual activity—acting as ERα antagonists in breast tissue but agonists in the bone and uterus—which is associated with an increased risk of endometrial cancer and thromboembolism. To address these risks, tamoxifen was succeeded by second-generation SERMs such as raloxifene, arzoxifene, and idoxifene, and third-generation agents like lasofoxifene, which offer improved bioavailability, fewer side-effects, and a reduced risk of thromboembolism (26).

In contrast, fulvestrant (ICI 182,780)—the only FDA-approved SERD for hormone receptor-positive (HR+) MBC—competes with E2 for ER binding with 89% of E2’s binding affinity, significantly higher than tamoxifen, which has only 2.5% of E2’s binding affinity (27). The fulvestrant–ER interaction impairs receptor dimerization, disrupts both activating function domains (AF1 and AF2) of ERα, inhibits energy-dependent nucleo-cytoplasmic trafficking, and accelerates ERα degradation (28). Unlike SERMs, fulvestrant lacks agonist activity in non-breast tissues and uniquely downregulates cellular levels of both ER and PR. However, its clinical efficacy is limited by poor bioavailability, suboptimal systemic exposure and biodistribution, and extensive hepatic metabolism via CYP3A4, necessitating intramuscular administration for controlled release (29, 30).

AIs, in contrast, work by disrupting estrogen biosynthesis and are classified into steroidal (type I), such as exemestane, and non-steroidal (type II), such as anastrozole and letrozole. These agents are widely used as adjuvant therapies for both early-stage and metastatic ER-positive breast cancer in postmenopausal women (31, 32). However, acquired resistance to AIs—often due to a switch from ER-dependent signaling to growth factor-mediated pathways—has led to the emergence of combination therapies (33, 34). Notably, pairing fulvestrant or AIs with CDK4/6 inhibitors has proven to be a promising and well-tolerated strategy for treating MBC. Recent clinical trials—PALOMA-3, MONALEESA-3, and MONARCH-2 (fulvestrant combined with palbociclib, ribociclib, or abemaciclib) (35–38), as well as PALOMA-2, MONALEESA-2, and MONARCH-3 (AIs combined with the same CDK4/6 inhibitors)—have demonstrated significantly improved progression-free survival (PFS) and overall survival (OS) compared to fulvestrant or AI monotherapy (39–41) Additionally, targeting the PI3K/AKT/mTOR signaling cascade with mTOR inhibitors, such as everolimus, represents a significant advancement in BC therapy (42, 43). In 2012, the FDA approved everolimus in combination with exemestane for the treatment of HR+ but HER2− breast cancer, providing an effective option for improving patient outcomes (44, 45). Despite the success of ET, acquired resistance develops in approximately 30%-50% of patients undergoing prolonged treatment, ultimately compromising therapeutic response and contributing to disease progression, metastasis, and relapse (46–49). Among the various factors, point mutations in the ERα ligand-binding domain (ERα−LBD) significantly contribute to the emergence of acquired resistance.

Recent deep DNA sequencing studies have identified activating mutations in the *ESR1* gene, which encodes ERα−LBD, in approximately 40% of recurrent, ET-resistant, ER-positive breast cancers (50–53). Most of these *ESR1* mutations are ligand-independent activation mutations that stabilize the unliganded ER in an agonist-bound conformation, thereby conferring constitutive activity and driving resistance to current ERα−targeted therapies. Among these, Y537S and D538G are the two most prevalent mutations (53, 54). Metastatic, therapy-resistant ER-positive breast cancers driven by *ESR1* mutations represent a significant clinical challenge and account for a substantial number of breast cancer-related deaths (55, 56). Deeper insights into the molecular mechanisms underlying mutant ERα activity is crucial for developing next-generation drugs targeting *ESR1* mutations with improved pharmacokinetic properties. In this context, several clinical trials are evaluating the safety and efficacy of next-generation SERDs—including elacestrant (RAD1901) (57, 58), camizestrant (AZD9833) (59), imlunestrant (LY3484356) (29, 60, 61), and giredestrant (GDC-9545) (62)—either as monotherapy or in combination with other anti-cancer agents, for targeting both *ESR1* wild-type and mutant ER+/HER2− locally advanced or MBC.

Other emerging therapeutic platforms, such as ER proteolysis-targeting chimeras (ER-PROTACs) like ARV-471 and ERD-3111 (63–66), complete estrogen receptor antagonists (CERANs) such as OP-1250 (Palazestrant) (67), and selective estrogen receptor covalent antagonists (SERCAs) like H3B-6545 (68), have demonstrated compelling preclinical anti-tumor efficacy and significant potency against clinically relevant ERα mutants, including Y537S and D538G. However, further studies are needed to evaluate long-term safety, side effect profiles, and recurrence prevention.

In parallel, GPER-mediated non-genomic signaling is emerging as a key contributor to ET resistance. Notably, the ability of both estrogen and anti-estrogens to activate GPER has led to findings that high GPER expression strongly correlates with tamoxifen resistance in BC patients (69–71). To address this, GPER-selective antagonists—such as G15 and G36—are being developed, offering further insights into the role of GPER in ER-positive breast cancer (72). This review emphasizes the structural features of ERs, particularly how the structure-function relationship of the ERα−LBD governs receptor activity, the role of activating *ESR1* mutations in driving constitutive signaling, and the development of next-generation therapeutics—especially those targeting ERα mutants and GPER—to simultaneously antagonize both receptor classes implicated in ET resistance.

## 2 Structure of ERs

**Figure 1:**
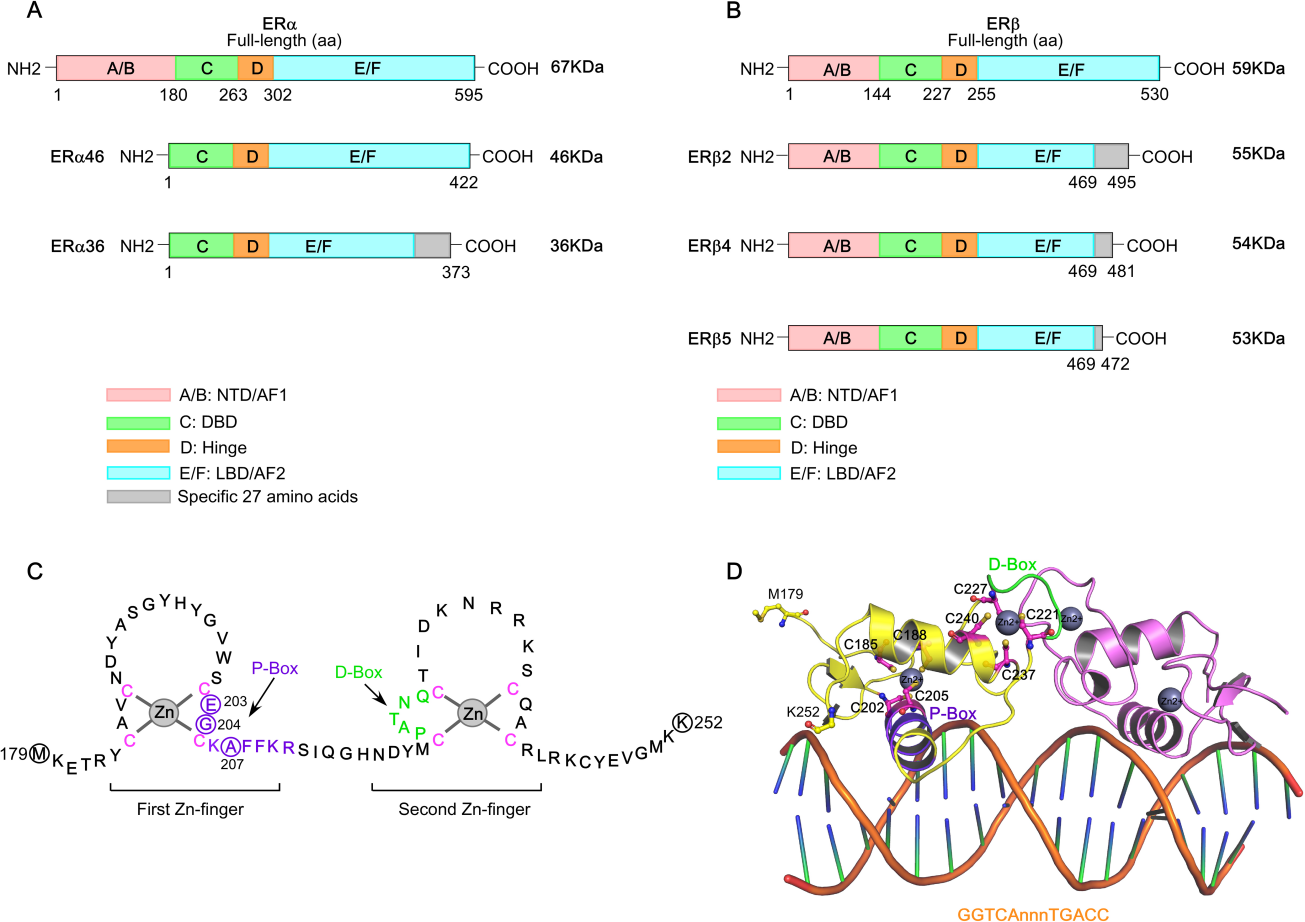
Structure of estrogen receptors (ERs). **(A)** Schematic figure of the structure of full-length ERα and its isoforms, ERα46 and ERα36. **(B)** Schematic figure of the structure of ERβ and its isoforms, ERβ2, ERβ4, and ERβ5. The domains of ERα and ERβ structures are color coded. **(C)** The DNA-binding domain (DBD) of ERα (Met179-Lys252). The ERα−DBD features two zinc-finger modules, each coordinated by a zinc ion (Zn2+) and four cysteine residues (indicated in pink). The amino acid residues forming the P-box and D-box are indicated in purple and green, respectively. **(D)** The cartoon structure of ERα−DBD dimer bound to consensus DNA sequence, GGTCAnnnTGACC (estrogen response element), with three non-specific (n) intervening bases (PDB: 1HCQ).

ERα, a 66 kDa protein composed of 595 amino acids, belongs to the nuclear hormone receptor (NHR) subfamily and is encoded by the *ESR1* gene located on chromosome 6 (6q25.1). The *ESR1* gene spans approximately 300 Kb and includes 8 exons that encode the full-length ERα protein (73). Structurally, ERα possesses conserved domains, including the N-terminal domain (NTD, ‘A/B’ domain), DNA-binding domain (DBD, ‘C’ domain), flexible hinge region (‘D’ domain) and ligand-binding domain (LBD, ‘E’ domain), followed by a short ‘F’ region (**Figure 1A**) (74–82). Two activation function domains, ligand-independent activation function (AF1) and ligand-dependent activation function (AF2), are located within the NTD and LBD, respectively, and mediate ER’s transcriptional activity. Alternative splicing of the *ESR1* gene generates an exon-1-truncated ERα transcript, ERα46, which lacks the N-terminal 1–173 amino acids, including the AF1 domain, and acts as a dominant-negative inhibitor of full-length ERα (83–85). Additionally, another isoform, ERα36, lacks both the AF1 and AF2 transactivation domains but retains a unique 22-amino acid C-terminal sequence (86). Interestingly, ERα46, expressed in various normal and tumor cell types including BC, contributes to cancer cell growth arrest by interfering with the binding of ERα66 to DNA (84, 87, 88). However, its expression is diminished in tamoxifen-resistant breast cancer cells, and re-expression of ERα46 suppresses cell proliferation and ERα66-regulated signaling (88, 89). Although an earlier study reported that the ERα46/ERα66 expression ratio is negatively correlated with breast tumor grade, a recent investigation highlighted a cross-talk between ERα46 and insulin receptor (IR) signaling that promotes the growth and pulmonary metastasis of the naturally immortalized BCAHC-1 cell line. Notably, this cell line—derived from a patient with invasive ductal breast carcinoma—exhibits unique co-expression and bi-directional cooperation between ERα46 and IR. This receptor cross-talk activates interleukin-11 (IL-11) expression and function, promoting the expression of pro-tumorigenic genes such as *ITGA5* and *ICAM-1*, and enhancing the migratory and invasive features of patient-derived breast cancer-associated fibroblasts (CAFs) (90). In contrast, tamoxifen acts as an agonist for ERα36 in breast cancer, enhancing stemness by upregulating ALDH1A1 and promoting ET resistance and metastasis (91).

The *ESR2* gene (spanning approximately 254 Kb), located on chromosome 14q23.2, encodes multiple ERβ isoforms due to alternative splicing or exon deletions of the last coding exon (exon 8), resulting in C-terminal truncations (92, 93). The full-length ERβ1 (60 kDa protein with 530 amino acids) is the only isoform capable of ligand-binding, whereas truncated isoforms such as ERβ2−β5 exhibit impaired ligand-binding activity due to the loss of AF2 function (**Figure 1B**) (94, 95). However, studies on ERβ isoform mRNA expression in breast cancer remain limited. Existing literature on the protein expression of different ERβ isoforms presents conflicting findings—some studies associate ERβ with favorable prognosis, while others report links to poor prognostic markers and reduced response to tamoxifen. Notably, ERβ2 mRNA expression is significantly correlated with better clinical outcomes in ERα−positive and node-negative tumors. A recent study further highlights that ERβ isoform mRNA and protein expressions are differentially associated with clinical characteristics and molecular subtypes of breast cancer (96). Simultaneous analysis of mRNA and protein expression levels of ERβ1, β2, and β5 across various BC subtypes revealed that ERβ isoform expression is significantly associated with Ki67 positivity (>15%), poor prognostic markers, and reduced OS. Specifically, high ERβ2 and β5 isoform expression is predictive of poor outcomes in ERα−negative breast cancer and TNBC.

**Figure 2:**
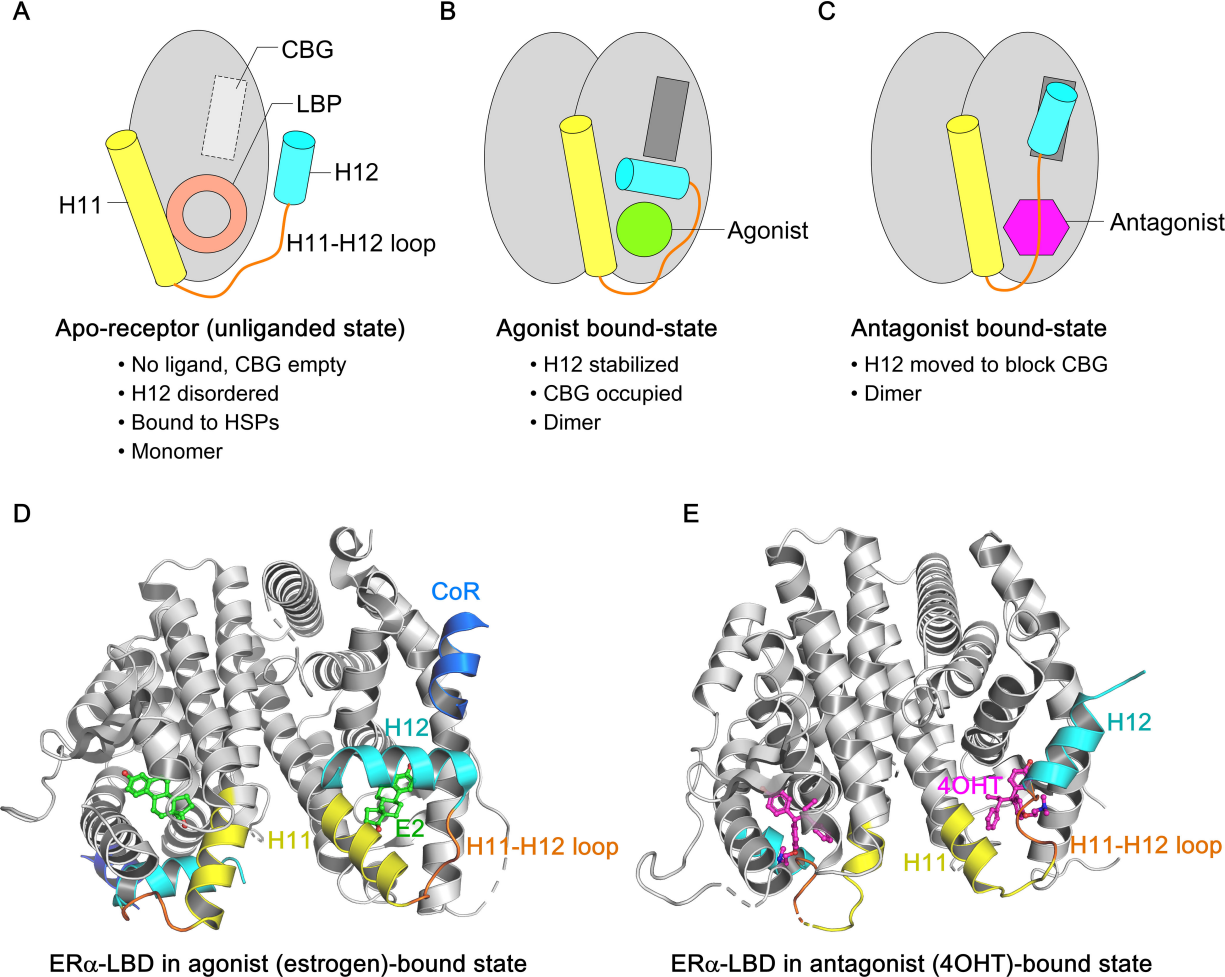
Overview of ligand-induced conformational states of ERα−LBD. **(A-C)** Schematic representation of three conformational states of ERα−LBD, highlighting the relative positions of H11 (yellow) and H12 (cyan) helices in the apo-state (no ligand), agonist-bound state (agonist in green), and antagonist-bound state (antagonist in purple), respectively. The H11–12 loop is shown in orange. **(A)** In the apo-state, both the ligand-binding pocket (LBP) and co-activator binding groove (CBG) are empty, preventing ER signaling. **(B)** In the agonist-bound state, H12 folds back to cover the LBP, enabling co-activator access to the CBG and initiating ER signaling. **(C)** In the antagonist-bound state, H12 shifts to block the CBG, inhibiting ER signaling. **(D)** The cartoon structure of wild-type ERα−LBD in complex with the agonist estrogen (in green sticks) and coregulator peptide (in blue) (PDB: 1GWR). **(E)** The cartoon structure of wild-type ERα−LBD in complex with the antagonist 4OHT (in purple sticks) (PDB: 5W9C). H11 and H12 helices are highlighted in yellow and cyan respectively, and the H11–12 loop in orange.

The NTD, DBD, hinge, and LBD of ERα and ERβ share 17%, 97%, 36%, and 56% amino acid identity, respectively (97). Full-transcriptional activity of ERα is achieved through the synergism of AF1 and AF2, where AF1 is hormone-independent and mediates constitutive activation, while AF2 requires estrogen binding for activation. AF1 is activated by phosphorylation at Ser104, Ser106, Ser118, Ser167, and Ser305 via signaling pathways such as PI3K/AKT, PKA, MAPK, and CDK2/7. The ERα−DBD mediates interaction with the palindromic hexanucleotide sequence 5’-AGGTCAnnnTGACCT-3’ within estrogen response elements (EREs), with two ERα−DBD monomers binding to adjacent major grooves of the ERE. The ERα−DBD comprises two zinc ion (Zn)-binding motifs (98–102), each co-ordinated by four cysteine residues. The ‘P-box’ within the first Zn-finger module (Glu203, Gly204, and Ala207) defines DNA-binding specificity to the ERE, while the ‘D-box’ within the second Zn-finger module (Pro222, Ala223, Thr224, Asn225, Gln226) is essential for half-site spacing discrimination (**Figures 1C, D**) (97, 98, 100, 103–107). Following the DBD, the ‘D’ domain—also known as the hinge region—connects the DBD to the LBD and contains the nuclear localization signal (NLS), which becomes exposed upon estrogen binding. The ‘E’ domain encompasses the LBD, including the ligand-binding pocket (LBP), a dimerization interface, and sites for co-activator and suppressor interaction. The ERα−LBD (amino acids 304-554) has a globular structure with 12 α−helices (H1-H12). In the absence of a bound ligand (apo-receptor or unliganded state), the LBD is partially disordered or inactive, remains associated with heat shock proteins (HSPs, primarily HSP90), and is likely monomeric (**Figure 2A**) (108–110). Upon binding an agonist like estrogen (agonist-bound state), the LBD sheds the HSPs, dimerizes, and adopts a stabilized “agonistic conformation”. In this conformation, the terminal helix H12 folds over the LBP, creating a hydrophobic groove that facilitates co-activator binding (**Figure 2B**). By contrast, when an anti-estrogen like tamoxifen binds the LBD (antagonist-bound state), helix H12 repositions to block the co-activator binding groove, thereby inhibiting receptor activation (**Figure 2C**). Cartoon structures of ERα-LBD in the agonist (estrogen)-bound conformation (PDB: 1GWR) and the antagonist (4OHT)-bound conformation (PDB: 5W9C) are shown in **Figures 2D, E**, respectively. Notably, the nuclear export sequence (NES) is located within the DBD and LBD of ERα. Following ‘E’ domain, both ER isoforms contain an unstructured carboxyl-terminal ‘F’ region, which shares only 18% amino acid identity. Recent advancements in cryo-electron microscopy (cryo-EM) have significantly advanced our understanding of ERα transcriptional complexes, offering detailed architectural insights that overcome the limitations of traditional structural and biochemical approaches (111–113). Single-particle cryo-EM analyses have elucidated how the functional domain organization of ER supports its interaction with core-coactivators and how these co-activators collaborate to modify histones and initiate transcription. The structural organization of the ER/co-activator complex reveals that ERα recruits two SRC-3 molecules (SRC-3a and SRC-3b), each interacting with distinct regions of p300, thereby facilitating the recruitment of p300 to the ERα−binding site (111). Importantly, dimer formation is a pre-requisite for ERα function, and mutations that disrupt ERα dimerization render the receptor transcriptionally inactive.

**Figure 3:**
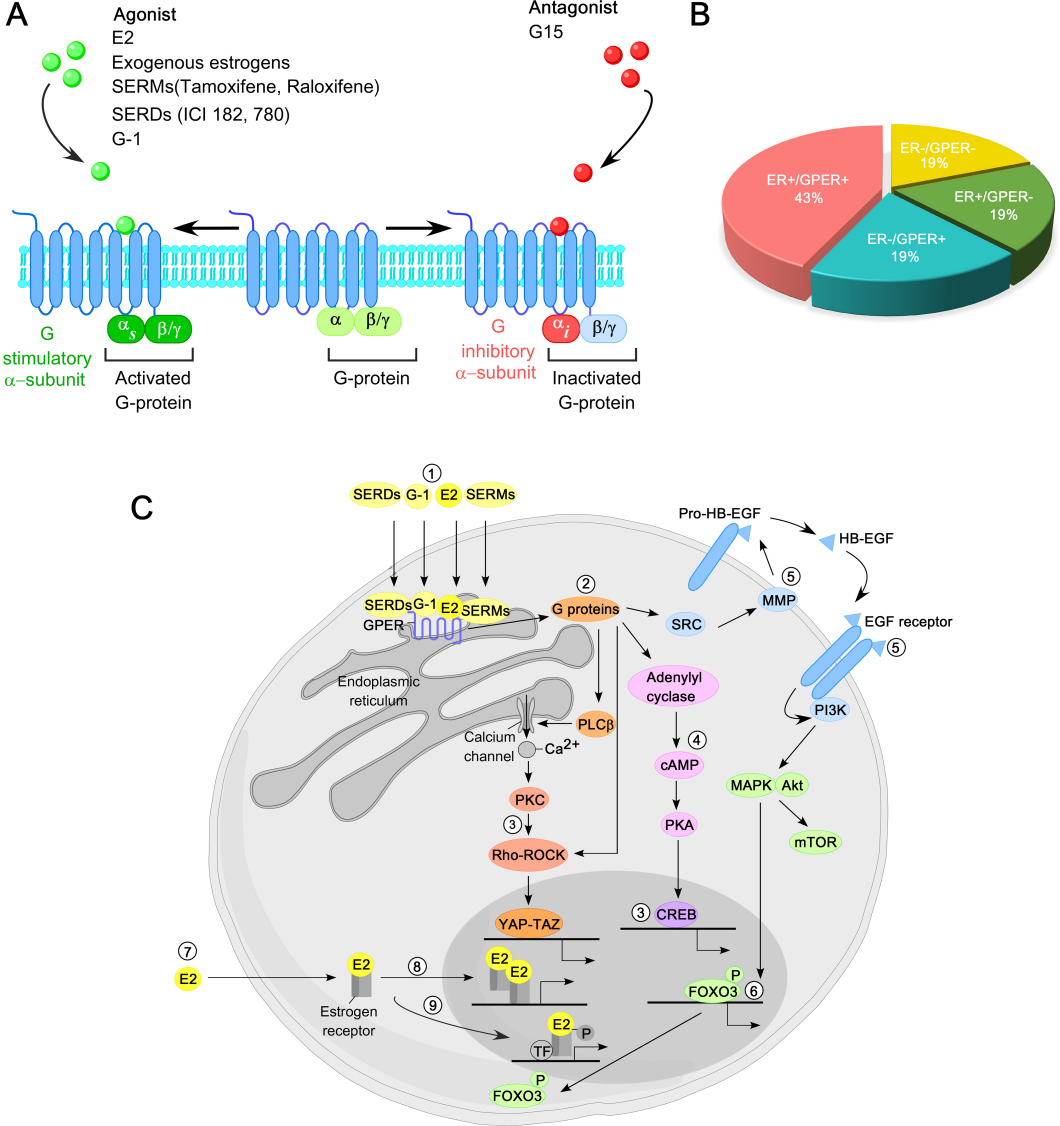
Overview of GPER function in breast cancer. **(A)** GPER is predominantly localized to the plasma membrane, featuring seven transmembrane helical domains, a ligand-binding pocket (LBP), and a G protein binding site. Upon interaction with estrogen or an agonist in its LBP, GPER activates a stimulatory G protein α−subunit (Gα*_s_*), leading to GPER activation. In contrast, interaction with antagonists triggers an inhibitory G protein α−subunit (Gα*_i_*), resulting in the inactivation of GPER. **(B)** Distribution of breast cancer based on the presence of ER and GPER in biopsy specimens. **(C)** Principal molecular pathways mediated by GPER in breast cancer. 17β−estradiol (E2), selective agonists such as G-1, SERMs, and SERDs activate GPER (1). GPER, in turn, activates heterotrimeric G proteins (2), triggering multiple downstream signaling cascades, including calcium mobilization from intra-cellular stores, activation of YAP-TAZ transcription factors via Rho/ROCK pathways (3), activation of Adenylyl cyclase-cAMP-PKA pathway (4), and activation of matrix metalloproteinases (MMPs) that cleave pro-heparin-binding epidermal growth factor (pro-HB-EGF) to release free HB-EGF, leading to EGFR trans-activation (5). This, in turn, activates MAPK (ERK1/2), Akt, and other signaling pathways. Activation of MAPK and Akt regulates gene transcription, including FOXO3 phosphorylation and degradation (6). In contrast, in the classical ER signaling, E2 binds to cytosolic or nuclear ERs (7), inducing receptor dimerization and binding to the promoter of ER-target genes (8). Additionally, activated ERs modulate the activity of other transcription factors (TFs) through protein-protein interactions (9).

The topology of GPER is highly conserved and consists of an N-terminal extracellular domain, seven transmembrane α−helical regions connected by three extracellular loops and three intracellular loops, and a C-terminal intracellular domain (114). The N-terminal domain is essential for receptor maturation from the endoplasmic reticulum (ER) to the plasma membrane (PM). The *GPER1* gene, located on chromosome 7 (7p22.3), encodes a 375-amino-acid protein with a molecular mass of 41 kDa. Upon binding ligands—including E2, SERMs, SERDs, or the GPER-selective agonist G-1—at either the extra-cellular surface or within the trans-membrane helices, GPER signals through a heterotrimeric G-protein. Estrogen or agonist binding activates the stimulatory Gα*_s_* subunit, thereby stimulating GPER, whereas antagonist binding activates the inhibitory Gα*_i_* subunit, leading to GPER inactivation (**Figure 3A**). Notably, both tamoxifen and fulvestrant exhibit significant binding affinity for GPER and can activate it in breast cancer. Interestingly, 43% of breast cancer biopsy samples co-express ER and GPER (**Figure 3B**). Moreover, physical interactions between GPER and both full-length ERα and ERα36 have been reported, suggesting a potential GPER-binding module in the ‘hinge’ region of both ERα (residues 295-311) and ERα36 (residues 123-139) (115, 116).

## 3 ERα Post-translational modifications: defining stability and nucleo-cytoplasmic dynamics

**Figure 4:**
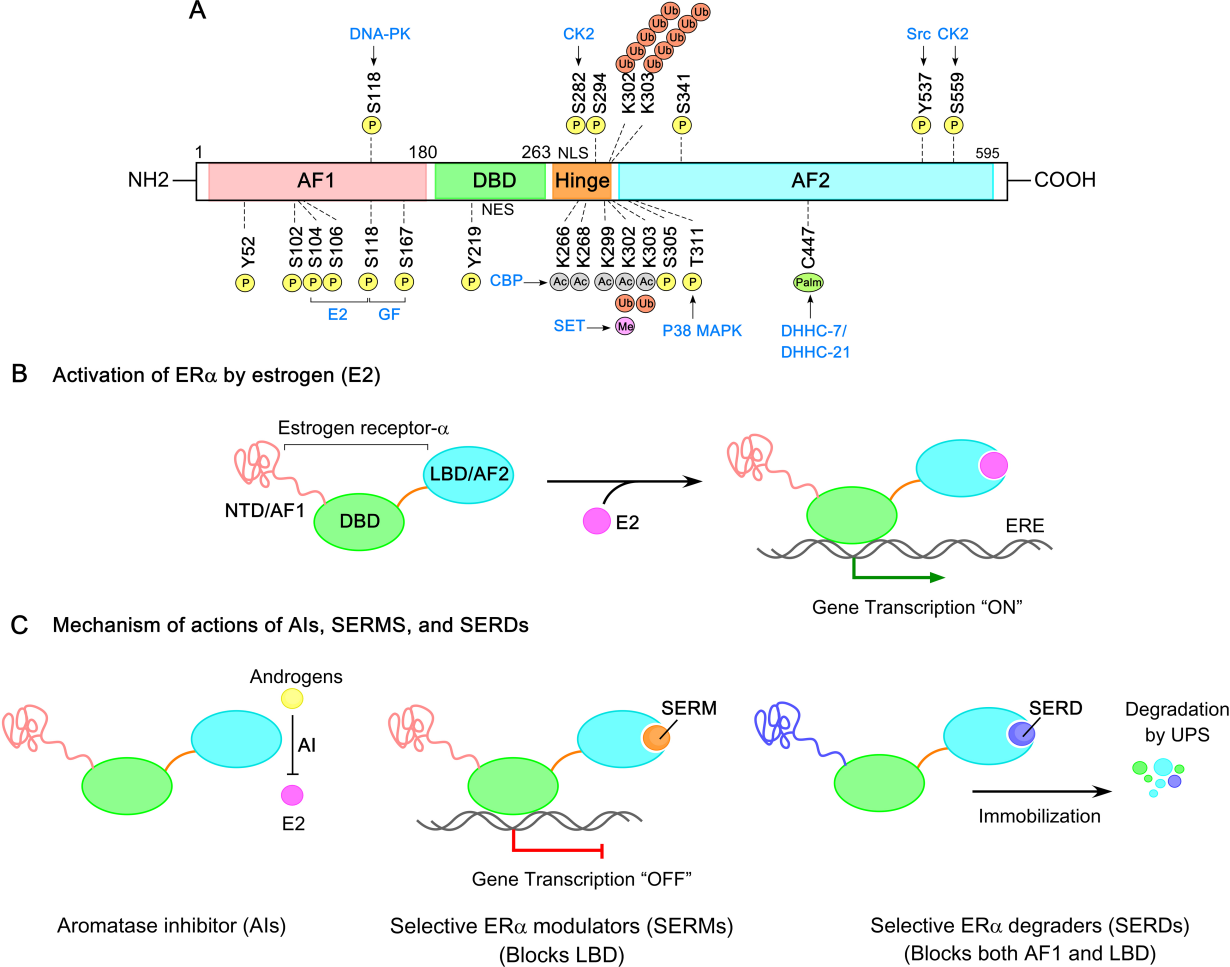
ERα Post-translational modifications (PTMs) and signaling pathways in breast cancer. **(A)** Amino acid residues in ERα subjected to phosphorylation, acetylation, palmitoylation, methylation, and ubiquitination are shown. Distinct post-translational modifications (PTMs) are color coded for clarity. **(B)** Estrogen-activated ERα initiates a transcriptional program that regulates target gene expression. **(C)** The mechanism of actions of aromatase inhibitors (AIs), selective ER modulators (SERMs), and selective ER degraders (SERDs). AIs block the conversion of androgens to estrogens, thereby reducing estrogen levels. SERMs inhibit the ERα−ligand binding domain (ERα−LBD) without affecting the DBD and AF1 domains. SERDs target both AF1 and LBD domains of ERα, leading to receptor immobilization, destabilization, and degradation.

Post-translational modifications (PTMs) of ERs, particularly ERα, play a crucial role in regulating its transcriptional activity in breast cancer and are fundamental to understanding ER biology (117). ERα undergoes PTMs under both ligand-dependent and ligand-independent conditions, often initiated by interactions with E2 or other ligands. The development of site-specific antibodies targeting post-translationally modified forms of ERα, along with advances in mass spectrometry, has greatly facilitated the identification of these PTM sites (118). To date, approximately 22 distinct PTM sites have been identified across the ERα structure, including phosphorylation, acetylation, sumoylation, and ubiquitination (**Figure 4A**). These modifications influence ERα’s stability (half-life), dimerization, transcriptional activity, subcellular localization, interactions with DNA and co-regulators, and degradation. In breast cancer cells, ERα is distributed across the cytoplasm and nucleus. Upon ligand binding (E2) to the ERα−LBD, ERα undergoes homo-dimerization and translocates to the nucleus, where the E2−ERα complex binds to EREs response elements (EREs) in the promoter regions of target genes. This binding facilitates co-regulator recruitment to the AF1/AF2 domains of ERα, driving gene expression. The schematic of ERα activation by estrogen is illustrated in **Figure 4B**.

Phosphorylation is a critical PTM of ERα, primarily targeting serine, threonine, and tyrosine residues. Among these, serine residues—particularly clustered within the N-terminal AF-1 region—are most frequently phosphorylated by MAPK, PI3K/AKT, and GSK-3, enabling ligand-independent transactivation of ERα. Key phosphorylation sites include Ser102, Ser104, Ser106, Ser118, Ser154, Ser167, Ser236, Ser294, Ser305, Ser559, Tyr52, Tyr219, Tyr537, and Thr311 (**Figure 4A**). Notably, Ser118, Ser167, and Ser305 are closely associated with ligand-independent ERα trans-activation and are often implicated in therapy-resistant ER-positive breast cancer.

Thomas et al. evaluated the relative significance of phosphorylation at Ser104, Ser106, and Ser118 for ERα activity, reporting the order of importance as Ser118>Ser104>Ser106 (119). Interestingly, substituting these serine residues with alanine had little effect, while replacement with glutamic acid (mimicking phosphorylation) markedly enhanced ERα activity, with the order of activity reversed—Ser106>Ser104>Ser118. Importantly, phosphorylation at Ser104/106 and Ser118 is essential for tamoxifen’s partial agonist activity, which has been linked to resistance in some breast cancers. Tamoxifen, exhibiting a dual role, inhibits the function of the LBD/AF-2 domain (antagonistic role) while simultaneously promoting ligand-independent activation of the AF-1 domain (agonistic role). The phosphorylation of Ser104/106 is estrogen-induced and mediated by kinases such as glycogen synthase kinase-3 (GSK3), cyclin-dependent kinase 2 (Cdk2), and MAPK (120). In contrast, Ser118 mediates both ligand-dependent and ligand-independent ERα activation, facilitating interactions with co-activators such as SRC-1 and CBP/p300, and is essential for ERα dimerization and RNA splicing (121–126). While estrogen induces Ser118 phosphorylation via kinases such as GSK3, IKKα, and CDK7, other stimuli—including epidermal growth factor (EGF) and insulin-like growth factor-1 (IGF-1)—can also trigger this modification through Ras-MAPK signaling. Recently, Du et al. showed that Ser118 phosphorylation triggers an unexpected conformational expansion of the intrinsically disordered ERα N-terminal domain (ERα−NTD), disrupting hydrophobic clustering between two aromatic-rich regions and promoting ligand-independent ERα activity (75, 127).

Phosphorylation of ERα at Ser305, mediated by protein kinase A (PKA) and p21 activated kinase 1 (PAK1), has been demonstrated to affect ER conformation, dimerization, interaction with coregulators, and DNA binding. Michalides et al. showed that this modification alters ERα conformation, contributing to tamoxifen resistance by preventing the receptor from adopting an inactive state despite tamoxifen binding (128). This conformational arrest shifts tamoxifen’s role from antagonist to agonist, promoting ERα−dependent transactivation. A phospho-mimetic ERα mutant, S305E, which mimics the constitutively phosphorylated state, exhibits increased binding to target gene promoters in the absence of ligand, suggesting that phosphorylation at Ser305 enables ligand-independent ERα activity (129). Thus, targeting PKA or blocking Ser305 phosphorylation offers a potential strategy to overcome endocrine resistance in breast cancer.

Conversely, phosphorylation at Ser167 is linked to favorable outcomes, including lower tumor grade, lymph node negativity, and longer relapse-free survival in BC patients (130–133). It also serves as a predictive marker for endocrine therapy response (134). In contrast, phosphorylation of ERβ remains less understood, with most identified sites located in the AF1 domain and the corresponding kinases yet to be identified.

Acetylation of ERα is a critical regulatory mechanism influencing its activity. ERα is acetylated by p300/CBP at five lysine residues—K266, K268, K299, K302, and K303 (**Figure 4A**) (135). Acetylation at K266 and K268 is estrogen-dependent and stimulatory, while modifications at K299, K302, and K303 are constitutive and suppress ERα transcriptional activity. Notably, the breast cancer susceptibility gene *BRCA1* inhibits ERα acetylation by blocking p300 binding to ERα acetylation sites and/or by mono-ubiquitinating ERα at K302. Consequently, *BRCA1* mutations increase the risk for BC development, while mutations at ERα acetylation sites—such as K266/268—confer resistance to *BRCA1*-mediated repression (136). Interestingly, K303 is a PTM hotspot, also subject to sumoylation and ubiquitination, and regulates methylation at adjacent K302. A recurrent K303R mutation, observed in ductal hyperplasia and invasive breast tumors, correlates with reduced relapse-free survival and confers resistance to tamoxifen and AIs by enhancing estrogen sensitivity (137–139). This mutation impairs K303 acetylation and promotes Ser305 phosphorylation. Barone et al. further showed that stable expression of a double K303R/S305A mutant receptor in MCF-7 cells induces AI resistance (137). Additionally, SET7-mediated methylation at K302 stabilizes ERα and enhances DNA binding, though acetylation at this site can hinder subsequent methylation (140). Notably, no acetylation sites have been identified for ERβ.

Palmitoylation — the reversible addition of palmitic acid to cysteine residues—regulates ERα stability, localization, activity, and membrane trafficking. ERα is palmitoylated at Cys447 by the acyltransferases DHHC-7 and DHHC-21 (**Figure 4A**), enhancing its hydrophobicity and anchoring it to membrane microdomains where it interacts with signaling molecules like Src (141, 142). This modification induces conformational changes that expose Src-binding sites, triggering rapid non-genomic estrogen signaling and promoting breast cancer cell proliferation. Upon E2 binding, ERα is depalmitoylated by acyl-protein thioesterases, leading to its dissociation from the membrane and translocation to the cytoplasm or nucleus. The dynamic palmitoylation-depalmitoylation cycle tightly regulates ERα function and represents a promising therapeutic target in ERα−positive BC.

Additionally, sumoylation of the ERα hinge region by SUMO-1 regulates its transcriptional activity (143). Notably, K266, K268, K299, K302, and K303 have been identified as key ERα sumoylation sites. Correspondingly, the double mutant (K266R/K268R) and the triple or five-lysine mutants (3K/R or 5K/R) exhibit significantly reduced levels of sumoylation compared to wild-type ERα, resulting in diminished transcriptional activity (144). However, sumoylation of ERβ has not yet been reported in the literature.

Furthermore, altered O-glycosylation of ERα is frequently observed in the majority of BC tissues, particularly in ERα−positive subtypes, where upregulated N-acetylgalactosaminyltransferase 6 (GALNT6 or GalNAc-T6) enzymatic activity is noted. Deng et al. demonstrated that GALNT6-mediated O-glycosylation at Ser573 is crucial for ERα stability and its nuclear trafficking in breast cancer cells (145). Consequently, targeting GALNT6 enzymatic activity or disrupting the GALNT6/ERα interaction with membrane-permeable peptides presents a promising therapeutic approach for ERα−positive breast cancer.

Ubiquitination adds another layer of complexity to ERα regulation. like other steroid receptors, ERα is subjected to ubiquitination via the 26S proteasome system, which governs both basal (ligand-independent) and ligand-induced degradation (146–148). In breast cancer cells, ERα degradation occurs through three distinct pathways: unliganded, ligand-bound (e.g., E2), and fulvestrant/other SERD-bound states. In its unliganded state, ERα is remarkably stable, with a half-life of up to five days. However, dynamic interactions with HSPs, co-chaperones, and E3 ubiquitin ligases (e.g. MDM2) target ERα for degradation (149), ensuring steady-state levels in the cytoplasm and maintaining homeostasis. Upon E2 binding, ERα’s half-life dramatically drops to 3–5 hours (150), as ligand-bound receptors are rapidly degraded to facilitate new protein synthesis. In contrast, fulvestrant and other SERDs induce ERα degradation independently of transcriptional activity or new protein synthesis (151, 152). Fulvestrant disrupts the HSP90-ERα complex and immobilizes ERα in the nuclear matrix, leading to its degradation (153). Berry et al. identified Lys302/303 as critical ubiquitination sites that protects against basal ERα degradation while promoting efficient E2- and fulvestrant-induced receptor turnover in BC cells (154). Key players in ERα ubiquitination include E3 ubiquitin ligases such as E6-AP, MDM2, EFP (estrogen-responsive finger protein), as well as the 26S proteasome and co-activators like SRC-1 and SRC-3. In the context of ERβ ubiquitination, the carboxy-terminus of HSP70-interacting protein interacts with N-terminus of ERβ receptor, facilitating its ubiquitination and eventual degradation.

Regarding GPER post-translational modifications, a recent study suggests that human GPER1 undergoes N-glycosylation, with asparagine 44 (Asn44) in the N-terminal domain being essential for receptor structure and activity (155). Mutating Asn44 to isoleucine inactivates the receptor, demonstrating that N-glycosylation at this site is critical for proper receptor maturation and trafficking to the plasma membrane. In contrast, residues 1–42 of the N-terminal domain do not appear to play a significant structural or functional role.

## 4 Regulatory factors governing ERα stability

Recent studies have identified key regulators that prolong ERα protein stability by inhibiting its polyubiquitination and degradation, thereby promoting ERα target gene expression and enhancing breast cancer cell proliferation. These ERα−polyubiquitination inhibitor proteins (EPIPs)—including kinases, transcriptional co-regulators, E3 ubiquitin ligases, and deubiquitinases—are often overexpressed in BC tissues, contributing to sustained ERα signaling and tamoxifen resistance. Notable EPIPs such as LMTK3, GSK3, cABL, TRIM family proteins, RNF8, RNF31, SHARPIN, and SMURF1 stabilize ERα by preventing its degradation (156). Collectively, these factors not only maintain elevated ERα levels and activity in breast tumors but also drive disease progression and therapeutic resistance.

### 4.1 Kinases and endonucleases

Several kinases—including LMTK3, DNA-PK, CK2, GSK3, and cABL—phosphorylate ERα, enhancing its stability and transcriptional activity while preventing degradation. LMTK3, a key ERα regulator in breast cancer, stabilizes ERα via direct phosphorylation and promotes its transcription by inhibiting PKC, reducing AKT phosphorylation, and facilitating FOXO3 binding to the ESR1 promoter (157–159). DNA-PK phosphorylates ERα at Ser-118, crucial for receptor stability and BC proliferation, with its inhibition leading to rapid ERα degradation (160). CK2 phosphorylates ERα at Ser167, Ser282 and Ser559, with Ser282 phosphorylation notably contributing to long-term receptor stabilization (161). Additionally, the endonuclease FEN1, often upregulated in tamoxifen-resistant breast cancer, enhances ERα transcription by supporting transcription complex assembly, and its inhibition leads to proteasome-mediated ERα degradation (162).

### 4.2 E3 ubiquitin ligases

Certain E3 ubiquitin ligases, especially members of the tripartite motif (TRIM) family, play critical roles in regulating ERα protein stability in breast cancer, by catalyzing the transfer of ubiquitin from E2 ubiquitin-conjugating enzymes to ERα lysine residues. While ubiquitination typically targets proteins for degradation, it can also modulate protein function and stability. Several TRIM proteins—including TRIM3, TRIM11, and TRIM56—enhance ERα stability (163, 164), whereas TRIM8 promotes its cytoplasmic degradation (165). For instance, TRIM56 interacts with the AF-1 domain of ERα and promotes K63-linked polyubiquitination, stabilizing ERα while inhibiting degradation-associated K48-linked ubiquitination (166). TRIM11, often overexpressed in BC, similarly stabilizes ERα, and its depletion impairs tumor cell proliferation and migration (163). Beyond TRIM proteins, atypical E3 ligases such as RNF31, RNF8, and SHARPIN mono-ubiquitinate ERα, shielding it from proteasomal degradation and enhancing ERα signaling (167–170). Additionally, SMURF1, HOIL-1, and RNF181 stabilize ERα by either inhibiting K48-linked ubiquitination or promoting K63-linked poly-ubiquitination (156, 171, 172). These findings highlight the crucial role of E3 ligases in modulating ERα turnover and activity, offering potential therapeutic targets for disrupting ERα−driven BC progression.

### 4.3 Ca2+ binding proteins

ERα transcriptional activity depends on its interaction with calmodulin (CaM), a ubiquitous Ca2+ sensor. Mutation of CaM (CaM1234), which disrupts Ca2+ binding, reduces E2-induced ERα transactivation in MCF7 cells. The interaction is mediated by ERα residues 298-303, particularly Lys-302 and Lys-303, which protect ERα from degradation and enhance its stability (173, 174). Additionally, calcineurin—a Ca2+ dependent phosphatase highly expressed in ERα−positive breast cancer with poor endocrine therapy response—stabilizes ERα by dephosphorylating Ser294, thereby preventing its degradation (175). Targeting the Ca2+/calmodulin complex or calcineurin, therefore, offers a potential therapeutic avenue for ERα−positive breast cancer.

### 4.4 Deubiquitinases

Deubiquitinases (DUBs) are proteases that regulate protein turnover by removing ubiquitin chains from substrate proteins, thereby influencing ERα stability in breast cancer. Several DUBs have been identified as key stabilizers of ERα, contributing to tumor progression and therapy resistance. USP7 shows a positive correlation with ERα levels in BC tissues and directly interacts with ERα to promote its deubiquitination and stabilization (176). Similarly, USP15 inhibits K48-linked ubiquitination of ERα, preventing its degradation, whereas USP15 depletion sensitizes ERα−positive breast cancer cells to tamoxifen (177). USP35 also stabilizes ERα, reducing the efficacy of tamoxifen and fulvestrant in ERα−positive breast cancer cells (178). Other DUBs, including OTUD7B and MINDY1, are over-expressed in breast cancer and support ERα stability by removing and K11- and K48-linked ubiquitin chains, with OTUD7B expression being associated with poor prognosis (179–181).

### 4.5 Concentration-inducible ERα function

The balance between ERα stability and degradation has significant implications for BC progression and therapeutic response. Fowler et al. demonstrated that elevated ERα concentrations can lead to its constitutive activation, driving aberrant promoter occupancy and gene expression even in the absence of estrogen (182). This phenomenon, termed “concentration-inducible ERα function”, involves serine 104/106/118-independent AF-1 transactivation and promotes breast tumor growth independently of estrogen, suggesting that ERα can drive transcription through mechanisms distinct from classical ligand-binding and phosphorylation-dependent pathways (182). High ERα concentration is often associated with poor prognosis and endocrine resistance in BC.

Besides, with the increasing use of AIs, breast cancer cells adapt to a low-estrogen environment, developing resistance through long-term estrogen deprivation (LTED). LTED induces estrogen hypersensitivity or super-sensitivity, enabling cells to respond to estrogen at concentrations 2–3 logs lower than those required for wild-type cells, or to grow in the absence of estrogen altogether (183–186). Both adaptations are characterized by elevated ERα expression, enhanced Ser118 phosphorylation, and activation of ERK1/2 and PI3K pathways, ultimately compensating for low estrogen levels. Paradoxically, ET resistance can also arise from reduced ERα levels due to enhanced degradation, as ERα is the primary target of SERMs and SERDs. For example, the ubiquitin-binding protein CUEDC2 promotes ERα degradation via the proteasome pathway; consequently, malignant mammary tumors with high CUEDC2 expression under tamoxifen-resistant conditions exhibit low ERα levels (187). These findings underscore that both prolonged ERα stability and accelerated degradation can disrupt the effectiveness of ET, highlighting the need for precise regulation of ERα homeostasis to optimize therapeutic outcomes.

In summary, ERα stability is not governed by a single linear pathway but by a dynamic and interconnected regulatory network of PTMs, protein-protein interactions, cellular signaling pathways, and subcellular trafficking mechanisms. PTMs—such as phosphorylation, mono-/poly-ubiquitination, deubiquitination mediated by kinases, E3 ubiquitin ligases, and deubiquitinases—play central roles in regulating ERα’s half-life, localization, transcriptional activity, and therapeutic resistance. These modifications often compete for the same sites on ERα, such as K303, underscoring the complexity of this tightly controlled system. Several cellular signaling pathways—including PI3K/AKT/mTOR and MAPK/ERK, Src, NF-κB and Wnt/β-catenin—are integral to maintain ERα stability and activity. Numerous studies have shown that ERα stability and nuclear export are critical for modulating both its nuclear and extra-nuclear functions, ultimately influencing BC progression and response to ET. Several proteins protect ERα from degradation while also impacting its subcellular distribution. For instance, elevated expression of dynein light chain 1 (DLC1) promotes E2-induced nuclear accumulation of ERα, enhancing its transcriptional activity (188). Conversely, the ERα mutant Y537F, which cannot bind the exportin protein CRM-1, accumulates in the nucleus and exhibits increased transcriptional activity. Normally, phosphorylation at Tyr537 by Src facilitates ERα interaction with CRM-1, promoting its nuclear export and subsequent degradation; The Y537F mutation disrupts this process, leading to ERα nuclear retention and heightened signaling (189). Collectively, these findings highlight the importance of both stability/degradation dynamics and subcellular trafficking in ERα regulation and endocrine resistance.

## 5 Structural insights into ERα hot-spot mutations & endocrine resistance:

Endocrine resistance—either *de novo* or acquired—is a major cause of relapse in ER-positive breast cancer. It reflects the tumor’s ability to evade or counteract therapies targeting the ERα signaling pathway, including tamoxifen, fulvestrant, and AIs (190). The mechanisms of action of these agents are illustrated in **Figure 4C**. Acquired resistance is frequently driven by emerging *ESR1* mutations, noted in a significant proportion of patients with ER+ MBC (191, 192). Additionally, the increased proportion of therapy-resistant tumor-initiating breast cancer stem-like cells (BCSCs; CD44+CD24−/lowLineage−) contributes to treatment failure and poor survival, especially in tamoxifen-resistant tumors (193). Briefly, these resistant cells overexpress drug efflux transporters and display stem-like characteristics, including enhanced proliferation, increased mammospheres formation, upregulation of stemness-related proteins (OCT-4, SOX2, Nanog, CD133), and increased epithelial-mesenchymal transition (EMT) plasticity. Fulvestrant resistance is associated with activation of the MEK/ERK, NF-κB, EGFR, PI3K/AKT, and β-catenin pathways. In contrast, AI resistance—which affects over 20% of early-stage and most metastatic cases—is driven by both intrinsic (e.g., upregulation of FGFR, ERBB2, IGF1R, PI3K-AKT-mTOR, MAPK signaling) and extrinsic factors, including interactions with the tumor microenvironment (34).

**Figure 5:**
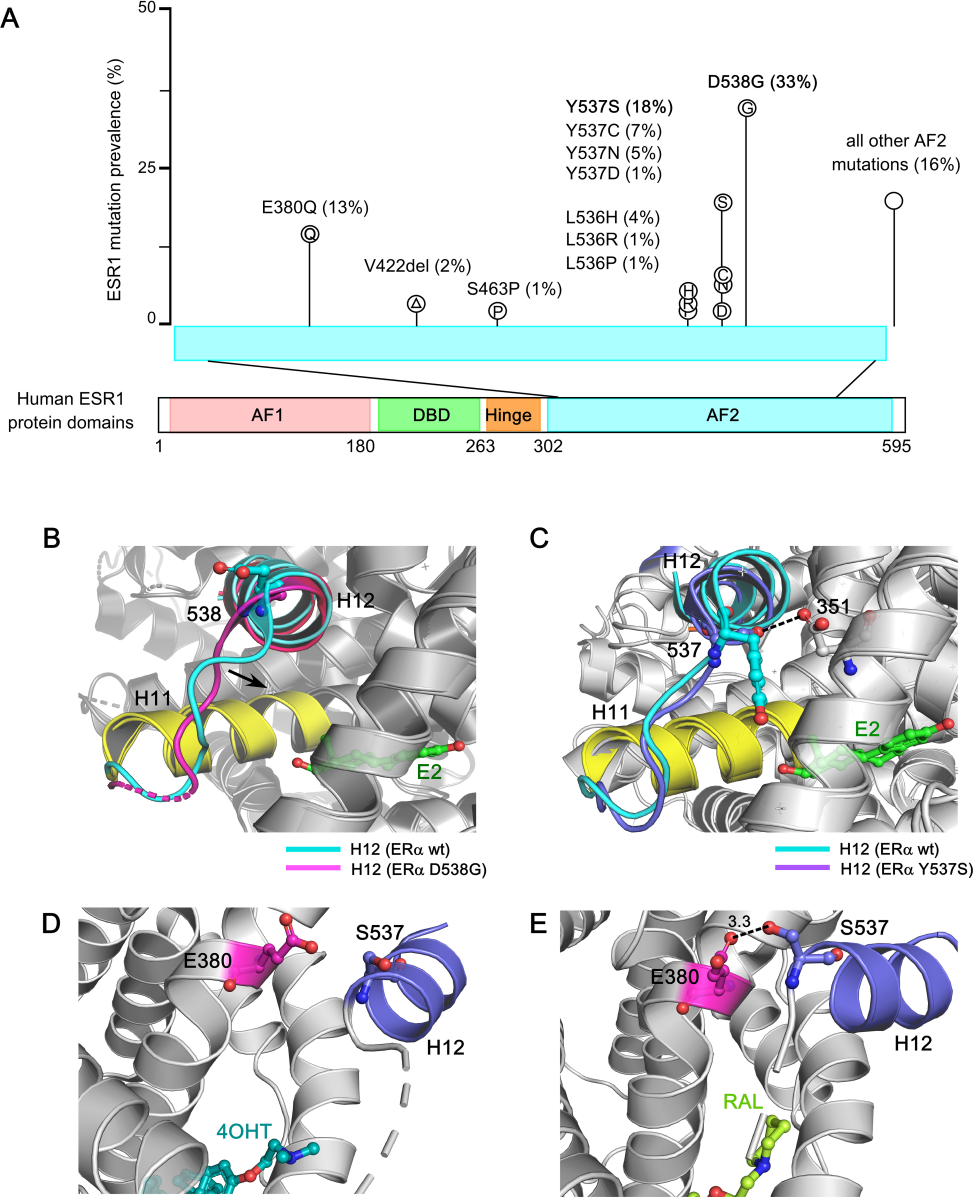
Structural basis of *ESR1* activating mutations and resistance to inhibition by SERMs and SERDs. **(A)** Prevalence of common *ESR1* mutations in breast tumor specimens from patients with endocrine-resistant ER+ breast cancer. The data is derived from two large retrospective studies, encompassing 2800 BC patients reflecting 283 *ESR1* mutations. **(B)** Superposition based on alpha carbons of wild-type ERα−LBD in complex with E2 (PDB: 1GWR) and D538G ERα (PDB: 4Q13). The H12 helix in wild type ERα and D538G ERα are highlighted in cyan and purple respectively. The H11 helix is highlighted in yellow for both the wild-type and mutant structure, and the ligand (estrogen) is shown in green sticks. The arrow denotes the direction of new H11-H12 loop packing into the hydrophobic hormone-binding pocket in D538G ERα mutant. **(C)** Superposition of alpha carbons from the wild-type ERα−LBD in complex with E2 (PDB: 1GWR) and the Y537S ERα mutant (PDB: 2B23), highlighting the S537-D351 hydrogen bond with a dashed line. The H12 helix in Y537S ERα mutant is shown in violet, and the Y537S mutation is shown in violet sticks. In Y537S ERα, the strong hydrogen bond between S537 and D351 lock the H11-H12 region in an agonist conformation, turning on constitutive activity. **(D)** The ineffective SERM 4OHT (in cyan-blue sticks) in complex with Y537S ERα mutant (PDB: 6V87). The H12 helix is shown in violet, and the S537 amino acid is highlighted in violet sticks. In complex with 4OHT, H12 helix is displaced from the AF2 cleft, enhancing co-regulator binding at AF2 and leading to ERα activation. **(E)** The effective SERM/SERD Raloxifene (RAL) (in light green) in complex with the Y537S ERα (PDB: 7UJC), stabilizes the antagonist conformation by forming a new S537-E380 hydrogen bond (3.3 angstrom). The hydrogen bond is indicated with a dashed line.

Large-scale genomic studies, such as The Cancer Genome Atlas (TCGA) project, have provided critical insights into the genomic landscape and heterogeneity of breast cancer, revealing a higher frequency of *ESR1* mutations in MBC. Constitutively active ERα mutants were first identified in the 1990s, through structure–function studies using random or site-directed mutagenesis of breast cancer cells in the absence of E2 or in the presence of anti-estrogens. Recent technological advancements, including next-generation sequencing (NGS) and droplet digital PCR (ddPCR), have enabled the detection of recurrent, missense, activating mutations clustered in ERα−LBD—particularly within the C-terminal H12 helix—in approximately 40% of BC patients previously treated with tamoxifen and AIs (51–53, 194–208). These activating ERα−LBD mutations are summarized in **Table 1**, including their proposed mechanisms of action, pharmacological phenotypes, and clinical implications. Since these mutations underscore the clinical need for more effective endocrine therapies, a detailed understanding of how the structure of ERα, particularly the ligand-induced conformation of its LBD, relates to its activity is essential (202). These mutations confer constitutive, ligand-independent activity at levels comparable to those induced by estrogen, implicating clonal selection as a key driver of endocrine resistance (48, 220, 221). Structural studies have shown that ERα−LBD mutations stabilize the receptor in an agonistic conformation, promoting ligand-independent ERα activation, altered gene expression, and changes in ERα−dependent cistrome (55, 222–224). The prevalence of common *ESR1* mutations in tumor specimens from patients with endocrine-resistant, ER-positive breast cancer is depicted in **Figure 5A**.

**Table 1:** Major ERα mutations, and their pharmacological phenotypes, mechanisms, and clinical impact.

Mutation site, zone, and frequency	Mutation category	Likely mechanism	Pharmacological phenotype	Clinical impact	References
D538G (H11–12 loop) ∼20% of patients with AI-treated MBC	Ligand-independent activation mutation, also classified under cofactor interaction altering mutation	“Lengthening” of H11–12 spring offers flexibility, enabling better sidechain packing for hydrophobic residues	Moderate constitutive activity, increased stability, increased affinity for co-activators in a ligand-independent way, relative AE resistance and more easily reversed by AEs	Increased BC cell proliferation and migration, E2-dependent PDX growth, increased tumor growth in mice	(51, 52, 202, 204, 207, 209)
Y537S, Y537N, Y537C, Y537D (H11–12 loop) ∼60% of mutations detected in MBC	Ligand-independent activation mutation; Y537S also classified under cofactor interaction altering mutation	Strong hydrogen bonding between S537 of Y537S mutant and D351 “latch” H11–12 spring in agonist-bound conformation	Increased stability and strong ligand-independent constitutive activity, reduced affinity for SERMs/SERDs due to pre-formed agonistic conformation, AE resistance in the following order: Y537S>Y537N∼Y537C, enhanced interaction with co-activators at AF2 cleft	Increased growth in cell-culture and mice, decreased CTC survival with HSP90 inhibitors or anti-HSP90/anti-AE treatment against Y537S ERα, increased cell proliferation with or without tamoxifen against Y537N ERα	(51, 52, 199, 202, 203, 207, 209–211)
L536R, L536H, L536P, L536Q (H11–12 loop) ∼1% of patients with AI-treated MBC	Ligand-independent activation mutation; also classified under cofactor interaction altering mutation	Replacing strongly hydrophobic leucine to charged (L536R), polar amino acid (L536H and L536Q), or less hydrophobic (L536P) reduces hydrophobicity, enabling rearrangement of H11–12 loop favoring ERα agonist conformation	Modest constitutive activity but harder to reverse with AEs, increased stability of L536P, increased binding to co-activators for ligand-independent activity	decreased CTC survival with HSP90 inhibitors or anti-HSP90/anti-AE treatment against L536P	(52, 202, 209, 212)
E380Q (H5 helix) ∼14% of patients with AI-treated MBC	Ligand-independent activation mutation	Neutralizes charge repulsion between acidic negatively-charged E380 in H5 and two acidic, negatively-charged residues (E542 and D545) in H12, favors an active ERα conformation without ligand binding	Modest constitutive activity, modest interaction with co-activators, enables the active conformation of ER without the energy provided by agonist ligand binding, require 3-times less E2 compared to wild-type ERα for achieving maximal activity	Enhanced ER signaling and target gene expression, BC cell proliferation in absence of E2, endocrine resistance	(53, 202, 209, 210, 213, 214)
K303R (at the border between Hinge domain and the beginning of LBD)	Ligand-independent activation mutation	Promotes phosphorylation at adjacent S305 by kinases, reduces ERα degradation by impairing ubiquitination	increased stability, prolonged receptor activity, enhanced binding to co-activators	Hypersensitivity to E2, increased cell growth in response to E2, confers resistance to tamoxifen and AIs, reduced relapse-free survival	(138, 209)
S463P (H9-H10 loop) 4% in MBC patients	Ligand-dependent activation mutation	Stabilizes ERα dimerization interface open for interaction and possibly enables additional intra-domain interaction, affects ERα binding to HSPs	Moderate constitutive activity, easily reversed by AEs, no interaction with co-activators without E2 stimulation *in vitro*, although E2-independent cell proliferation has been noted raising questions about its functional role in BC	NA	(202, 207, 209)
ERα−36 (lacks the AF-1 and large portion of the LBD)	ERα−LBD isoforms (truncated)	Mediates non-genomic oncogenic signaling in the presence or absence of ligand	NA	Increased BC cell survival and invasiveness, increased resistance to AEs, poor survival in BC patients, tamoxifen resistance	(210, 215–218)
ERα−46 (lacks AF-1 only)	ERα−LBD isoforms (truncated)	Mediates membrane-associated E2 signaling	NA	Functions as dominant-negative regulator inhibiting full-length ERα activity	(84, 210, 219)

E2, estrogen; CTC, circulating tumor cells; AE, anti-estrogen; AF2, activation function 2.

Importantly, the dynamic nature of the H12 helix plays a critical role following the E2: ERα−LBD interaction. Among the most prevalent point mutations in ERα, Tyr537 is the most frequently mutated site, giving rise to four distinct variants: Y537S, Y537N, Y537C, and Y537D. These mutations interfere with receptor degradation, contributing to ET resistance and metastasis in breast cancer patients. Hot-spot mutations in the ERα structure—such as Y537S, Y537N, Y537C, D538G, and E380Q—differentially impact its structural integrity, promoting estrogen-independent activity. The ERα−LBD is an intrinsically disordered α−helical bundle that encapsulates a hydrophobic LBP, where estrogen binds, and the AF2 domain, which serves as the interaction site for ligand-dependent co-regulators. Access of co-regulators to the AF2 cleft depends on the structural dynamics of H12 helix within the ERα−LBD (225). In the apo or unliganded state, the H12 helix is highly dynamic, rendering the AF2 site inaccessible to coregulators (see **Figure 2A**). Estrogen binding provides favorable folding energies, allowing H12 helix to fold over the LBP, thereby opening the AF2 cleft for co-regulator interactions (PDB: 1GWR) (226) (see **Figure 2B**). Furthermore, this interaction exposes a hydrophobic patch in the loop between H11 and H12, resembling a “spring-like strained conformation” stabilized by estrogen. Mutations at leucine-536 (L536), tyrosine-537 (Y537), and aspartate-538 (D538) relieve this tension by reducing the hydrophobicity of this patch, stabilizing the unliganded ERα in an agonist-bound conformation (227). The D538G mutation, in the H11-H12 loop of ERα−LBD, is observed in ∼20% of BC patients with AI-treated metastatic disease and causes the “lengthening” of the H11-H12 spring in ERα, conferring constitutive activity (**Figure 5B**). (228). In contrast, high-resolution x-ray crystal structure reveals that in the Y537S mutation, S537 establishes a new hydrogen bond with D351, stabilizing the H12 helix in an agonist-bound conformation (PDB: 2B23) (229) (**Figure 5C**). This mutation confers greater therapeutic resistance to 4OHT by enhancing co-regulator binding at the AF2 cleft (PDB: 6V87), leading to ERα activation (**Figure 5D**). However, raloxifene (RAL) in complex with the Y537S ERα−LBD mutant favors the highly buried H12 antagonist conformation through the formation of a new S537-E380 hydrogen bond, effectively turning the receptor off (**Figure 5E**). Interestingly, the Y537S and D538G mutants exhibit a 3-10-fold reduced affinity for SERMs/SERDs due to their pre-formed agonistic conformation, contributing to ET resistance. These mutants also drive transcriptomic reprogramming, resulting in increased expression of metastasis-related genes. Notably, the E380Q mutant requires three times less estrogen than wild-type ER to achieve its maximal activity, while the S463P mutation leaves the ERα dimerization domain constitutively open for interaction. Additionally, mutations at leucine-536 (L536H/R/P/Q) compromise the structural integrity of the receptor, causing it to adopt a ligand-bound active conformational state (228).

As dynamic biomarkers of disease progression and endocrine resistance, *ESR1* mutations present a valuable platform for improving clinical outcomes in ER-positive metastatic breast cancer. In this context, Goldberg et al. identified the most frequent *ESR1* mutations−Y537S, D538G, and E380Q−as novel targets for developing breast cancer immunotherapies aimed at restoring endocrine sensitivity (230). Notably, mutations such as Y537N/C/S and D538G have been detected in circulating tumor DNA (ctDNA) in 39.1% of metastatic patients, showing a strong correlation with resistance to AIs (231). Furthermore, long-term estrogen deprivation (LTED), as previously discussed, promotes the selection of naturally occurring *ESR1* mutations, including Y537C and Y537S, in *ESR1*-positive cell lines (221).

To further investigate the functional implications of these mutations, CRISPR-Cas9−engineered mutant breast cancer cell lines harboring L536R, Y537C, Y537N, Y537S, and D538G mutations demonstrated varying sensitivities to anti-estrogens such as tamoxifen and fulvestrant (227). Consistently, clinical data from the PALOMA-3 and MONARCH-2 trial control arms showed that fulvestrant was less effective in patients with *ESR1* mutations compared to those with wild-type ER, highlighting increased resistance in this subset (232, 233). These acquired *ESR1* mutations underscore the clinical need for developing next-generation ERα−targeted agents. Both the pharmaceutical industry and academia have been actively working to design novel ER inhibitors that block the ER signaling pathway, with each class operating through a distinct mechanism of action (see **Table 2**).

**Table 2:** Next-generation ER-targeting agents in clinical trials.

Endocrine agent	ET class and developing company	Study design	Patient characteristics	Clinical trial identifier (ER+ MBC)	References
Lasoxifene	SERM (oral) Sermonix	Monotherapy versus Fulvestrant	ER+/HER2− metastatic breast cancer with *ESR1* mutations	NCT03781063 (ELAINE 1)	(26, 234, 235)
		Combined with Abemaciclib versus Fulvestrant + Abemaciclib	ER+/HER2− Locally advanced or MBC with *ESR1* mutations	NCT05696626 (ELAINEIII)	
Bazedoxifene	SERM/SERD hybrid (oral) Pfizer	Palbociclib in combination with Bazedoxifene	Hormone-receptor positive breast cancer	NCT02448771	(236–238)
Elacestrant (RAD1901)	SERM/SERD hybrid (oral) Radius Health	Monotherapy versus SOC	ER+/HER2− advanced breast cancer with *ESR1* mutations	NCT03778931 (EMERALD)	(239, 240)
		Combined with Abemaciclib	Brain metastasis due to HR+/HER2− breast cancer	NCT05386108 (ELECTRA)	
Camizestrant (AZD9833)	SERD (oral) Astra Zeneca	Combined with CDK 4/6 inhibitors versus AI+CDK 4/6 inhibitors	HR+/HER2− MBC with detectable *ESR1* mutation	NCT04964934 (SERENA-6)	(241, 242)
Giredestrant (GDC-9545)	SERD (oral) Genentech/ Roche	Combined with Palbociclib versus Letrozole + Palbociclib	ER+/HER2− locally advanced or MBC	NCT04546009 (persevERA)	(243–247)
		Combined with Everolimus versus ET + Everolimus		NCT05306340 (evERA)	
Imlunestrant (LY3484356)	SERD (oral) Eli Lilly	Monotherapy and combined with Abemaciclib/Everolimus/Alpelisib	ER+/HER2− locally advanced or MBC	NCT04188548 (EMBER)	(60, 61, 248)
		Monotherapy and combined with Abemaciclib		NCT04975308 (EMBER-3)	
Rintodestrant (G1T48)	SERD (oral) G1 Therapeutics	Monotherapy and combined with Palbociclib	ER+/HER2− MBC	NCT03455270	(249, 250)
Borestrant (ZB-716)	SERD (oral) Zeno Pharma	Monotherapy and combined with Palbociclib	ER+/HER2− locally advanced or MBC	NCT04669587 (ENZENO)	(251, 252)
Taragarestrant (D-0502)	SERD (oral) Inventisbio	Monotherapy and combined with Palbociclib	ER+/HER2− advanced or MBC	NCT03471663	(253, 254)
LX-039	SERD (oral) Louxin Pharmaceutical	Dose escalation and dose expansion	ER+/HER2− locally advanced or MBC	NCT04097756	(255, 256)
ZN-c5	SERD (oral) Zentalis	Monotherapy and combined with Palbociclib	ER+/HER2− advanced breast cancer	NCT03560531	(257–259)
		Combined with Abemaciclib		NCT04514159	
H3B-6545	SERCA (oral) H3 Biomedicine	Combined with Palbociclib	ER+/HER2− locally advanced breast cancer or metastatic breast cancer	NCT04288089	(260–262)
Palazestrant (OP-1250)	CERAN (oral) Olema Oncology	Combined with Palbociclib	ER+/HER2− advanced or MBC	NCT05266105	(263–265)
		Combined with Ribociclib and Alpelisib		NCT05508906	
		Monotherapy versus SOC ET (Fulvestrant, anastrozole, letrozole, or exemestane)		NCT06016738 (OPERA-01)	
Vepdegestrant (ARV-471)	PROTAC (oral) Arvinas	Monotherapy versus Fulvestrant	ER+/HER2− advanced or MBC	NCT05654623 (VERITAC-2)	(266–269)
		Combined with Palbociclib versus Letrozole + Palbociclib		NCT05909397 (VERITAC-3)	
AC699	Chimeric ER Degrader (oral) Accutar Biotech	Safety, tolerability, PK, and anti-tumor efficacy	ER+/HER2− advanced or MBC	NCT05654532	(65, 270)

Importantly, the absence of detectable *ESR1* mutations in primary breast tumors suggests that these mutations emerge through clonal selection during tumor evolution, enabling tumor cells to evade hormonal therapies. To monitor such adaptive genomic alterations, single-cell DNA sequencing of both tissues and serial plasma samples could enable real-time tracking of *ESR1* mutation dynamics across disease stages. Early detection of *ESR1* mutations in subclonal populations may help optimize adjuvant therapy decisions. Additionally, structural modeling of mutant ER could provide insight into conformational alterations and aid in designing peptide-based or alternative targeted therapies. Given the critical role of co-activators in the ligand-independent activity of mutant ERα, disrupting these interactions may represent a promising therapeutic strategy to reverse endocrine resistance.

## 6 Role of GPER in ERα–positive breast cancer

GPER is primarily localized to intra-cellular membranes, including the endoplasmic reticulum and Golgi apparatus, where it mediates non-genomic estrogen signaling (**Figure 3C**). In 2007, the International Union of Basic and Clinical Pharmacology officially designated GPR30 as GPER, recognizing it as a therapeutic target in breast cancer, including ERα−positive subtype (271–274). GPER is broadly expressed in breast cancer cell lines and primary tumors, with high expression levels correlating with increased tumor size, metastasis, tamoxifen resistance, and poor prognosis. Therefore, delineating ER−GPER crosstalk is crucial for understanding BC progression and ET resistance in ERα−positive tumors.

Notably, SERMs such as tamoxifen and raloxifene, and SERDs like fulvestrant, act as GPER agonists, inducing its expression and activating pro-survival signaling pathways (27, 69, 275–279). Due to GPER’s distinct pharmacological profile, the development of ERα-selective agents that do not cross-react with GPER is essential. Parallel efforts to develop GPER-selective ligands have deepened our understanding of its role in BC progression (see **Table 3**) (293). A notable example is G-1, a GPER-selective agonist identified through compound library screening in 2006 (280). Additional GPER- agonists include indole-thiazole derivatives such as GPER-L1 and GPER-L2 (282). The discovery of GPER-selective antagonists—G15 and G36 (290, 291)—has further illuminated GPER’s functions in breast cancer. Other antagonists include MIBE (Molecular Inhibitor for Breast Cancer Estrogen Receptor), pan-estrogen receptor antagonists, and CIMBA. MIBE targets both ERα and GPER, blocking their activation by estrogen and related agonists. Pan-estrogen receptor antagonists inhibit ERα, ERβ, and GPER, whereas G36 selectively targets GPER, blocking non-genomic signaling without significantly affecting ERα or ERβ. Its structural analogue, CIMBA, demonstrates even greater GPER-binding affinity and specificity (294). Two novel benzopyrroloxazine-based selective GPER antagonists, PBX1 and PBX2, inhibit GPER-dependent signaling in breast cancer cells and cancer-associated fibroblasts (CAFs), but require further validation in preclinical and clinical trials (295).

**Table 3:** GPER agonists and antagonists in breast cancer.

Name	Mechanism of action	Experimental cell lines	Specificity for GPER	References	
Agonists					
G1	Binds specifically to GPER and activates GPER/EGFR/ERK pathway	SKBR3, MDA-MB-453, HCC70, HCC1806	Specific	(280)	
17β-estradiol (E2)	Activates GPER, triggers rapid activation of GPER/EGFR/ERK pathway, driving BC proliferation and invasion	MCF-7, SKBR3, MDA-MB-231, MDA-MB-436, MDA-MB-468	Non-specific	(281)	
Tamoxifen	Binds to GPER and upregulates its expression, promotes BC proliferation, resulting in ET resistance	MCF-7, SKBR3	Non-specific	(275)	
ICI182,780 (fulvestrant)	Binds to GPER, activates ERK and PI3K pathway, resulting in endocrine resistance	MCF-7	Non-specific	(27)	
GPER-L1	Upregulates GPER-target genes, inducing BC proliferation	SKBR3	Specific	(282)	
GPER-L2					
27-hydroxycholesterol	Mediates activation of ERK1/2 and NF-κB, enhancing BC proliferation	MDA-MB-231, MDA-MB-468	Non-specific	(283)	
Bisphenol A (BPA)	Induces EGFR and FAK/SRC/ERK pathway, mediates BC migration	MDA-MB-231	Non-specific	(284)	
Bisphenol S (BPS)	Promotes TNBC metastasis through GPER/Hippo-YAP pathway	MDA-MB-231, BT-549	Non-specific	(285)	
Tetrachlorobisphenol A (TCBPA)	Upregulates GPER, mediates ERK/AKT signaling to promote BC proliferation	SKBR3, MCF-7, MDA-MB-231	Non-specific	(286)	
Chrysin-nanoparticles (NP)	NPs activate GPER and suppress PI3K, p-JNK, and NF-κB expression to inhibit TNBC proliferation and migration	MDA-MB-231	Non-specific	(287)	
Tanshinone IIA	Binds to GPER and promotes apoptosis in TNBC cells, inhibiting migration via GPER/EGFR/ERK signaling pathway	MDA-MB-231	Non-specific	(288)	
Berberine (BBR)	Promotes GPER transcription and inhibits viability and migration of breast cancer cells	MDA-MB-231, MDA-MB-436, MDA-MB-468	Non-specific	(289)	
Antagonists					
G-15	Inhibits GPER-dependent E2 signaling	HCC1806, HCC70	Specific	(290)	
G-36	Inhibits GPER-dependent E2 signaling	SKBR3	Specific	(291)	
Estriol (E3)	Inhibits GPER/EGFR/ERK signaling pathway and retards breast cancer cell proliferation	SKBR3	Non-specific	(292)	

Recent studies emphasize the prognostic significance of GPER localization: plasma membrane-localized GPER correlates with poor outcomes, while its absence on the plasma membrane is associated with excellent long-term prognosis in tamoxifen-treated tumors (296). Cytoplasmic GPER is linked to non-ductal histologic subtypes, better differentiation, and lower tumor grades, while nuclear GPER is associated with poorly differentiated carcinomas and TNBC subtypes (297, 298). These findings underscore the need for precision therapies tailored to GPER expression levels and subcellular localization in BC patients.

### 6.1 GPER and phyto- and xeno-estrogens molecules

A wide range of phytoestrogens and xenoestrogens stimulate cAMP production, activate protein kinases, and drive GPER-dependent gene transcription in BC cells. Phytoestrogens—such as quercetin (299), genistein (300, 301), resveratrol (302), (-)-epicatechin, oleuropein, daidzein (303), equol, and icariin—are plant-derived compounds that mimic estrogen and target ERs. In contrast, xenoestrogens are synthetic, chemically stable endocrine-disrupting chemicals (EDCs) found in plastics, surfactants, pesticides, and pharmaceuticals. Examples include Bisphenol A (BPA), polychlorinated biphenyls (PCBs), diethylstilbestrol (DES), and Dichlorodiphenyltrichloroethane (DDT) and its metabolites. These compounds often act as GPER agonists and interact with both classical ERs and GPER, sometimes exerting opposing effects (285, 304, 305). For instance, 4OHT functions as an ERα antagonist but a GPER agonist, whereas estriol (E3) acts as an ERα agonist but a GPER antagonist.

### 6.2 GPER-mediated non-genomic signaling in breast cancer

GPER-mediated non-genomic signaling elicits rapid cellular responses independent of direct gene expression (306). Upon activation by E2 or ER antagonists, GPER initiates intracellular signaling cascades at the plasma membrane, leading to the production of second messengers such as cAMP, IP3, DAG, and Ca2+. These molecules activate downstream kinases including PKA, PKC, and MAPKs (**Figure 3C**), which drive cell proliferation, migration, and invasion. GPER also regulates the expression of genes such as *c-FOS* (299), *CTGF*, and *EGR1*, promoting tumor progression. It enhances motility via cyclins (A1, D, E), CTGF, CXCR1, and Notch signaling. For example, Chen et al. demonstrated that estrogen and fulvestrant enhance MCF-7 adhesion to the extracellular matrix via the GPER-calpain axis (307). GPER activation also promotes invasion of inflammatory BC cells by activating p-ERK1/2, suggesting its role in metastatic dissemination (308). Importantly, GPER expression is higher in metastatic lesions than in matched primary tumors, underscoring its role in disease progression. In TNBC, GPER has strong prognostic value, particularly in aggressive subtypes, including basal-like, immunomodulatory, mesenchymal-like, and luminal androgen receptor (LAR). Elevated GPER expression is strongly associated with reduced relapse-free survival (RFS) and distant metastasis-free survival (DMFS), especially in patients with additional risk factors such as lymph node metastasis (LNM), high tumor grade (G3), and advanced TNM stage (309). Zhu et al. further demonstrated that GPER activation enhances TNBC cell stemness, increasing the CD44+CD24−/low population and upregulating stemness-related genes in MDA-MB-468-derived mammospheres (310). These findings support the therapeutic potential of GPER-targeted inhibitors in managing aggressive BC subtypes, including TNBC (311).

### 6.3 GPER & tamoxifen resistance in ERα–positive breast cancer

Elevated GPER levels have been observed in BC patients primarily treated with tamoxifen, linking GPER signaling to tamoxifen resistance (69, 312). Early studies demonstrated that 4OHT exerts GPER agonistic activity, potentially inducing tamoxifen-resistant tumors instead of inhibiting them (301, 313). Through sustaining estrogen signaling in the presence of tamoxifen, GPER contributes to ET resistance, with AIs proving more effective than tamoxifen in ER+/GPER+ tumors. Ignatov et al. further reported that tamoxifen-treated patients with GPER-positive tumors exhibited increased GPER expression and decreased OS compared to those who did not receive tamoxifen (69). Mechanistically, tamoxifen cross-activates GPER, inducing proliferation of resistant breast cancer cells and promoting the nuclear expulsion of the pro-apoptotic transcription factor FOXO3a, thereby shifting cells toward a pro-survival state (314). Additionally, tamoxifen-mediated GPER cross-activation increases aromatase expression, further exacerbating resistance (275). Preclinical evidence supports targeting GPER as a strategy to overcome tamoxifen resistance: GPER knockdown or co-treatment with the GPER antagonist G15 attenuates breast cancer cell proliferation (70), and combining G15 with tamoxifen restores sensitivity in tamoxifen-resistant MCF-7 xenografts. Furthermore, G15 sensitizes epithelial breast cancer cells to doxorubicin by inhibiting EMT through GPER down-regulation (315). Collectively, these findings highlight the complex interplay between GPER and ERα signaling in driving gene expression changes that fuel ERα−positive BC progression. The non-genomic pathways mediated by GPER, along with critical intermediates and enzymes involved, are outlined below (refer to **Figure 3C**):

### 6.4 GPER, IP3-dependent calcium mobilization, and activation of the YAP-TAZ pathway

Upon activation by E2, G-1, SERMs, or SERDs, GPER interacts with hetero-trimeric G-proteins (Gα, Gβ, and Gγ) on the inner surface of the plasma membrane (316). G-protein activation leads to the dissociation of Gαq/11 from the Gβγ dimer. Activated Gαq/11 then stimulates phospholipase C (PLC), which catalyzes the hydrolysis of PIP2 (phosphatidylinositol 4,5-biphosphate) into IP3 (inositol triphosphate) and DAG (diacylglycerol). IP3 binds to its receptors on the endoplasmic reticulum, triggering Ca2+ release into the cytosol (**Figure 3C**), while DAG activates protein kinase C (PKC). The rise in cytosolic calcium concentration activates calcium-dependent kinases such as calcium/calmodulin-dependent protein kinase II (CaMKII) and promotes actin cytoskeleton reorganization. Simultaneously, GPER signaling activates Rho-GTPases, including RhoA, enhancing actin cytoskeleton assembly and increasing cellular tension. This mechanical tension inhibits the Hippo pathway, allowing unphosphorylated YAP (Yes-associated protein) and TAZ (transcriptional coactivator with PDZ-binding motif) to translocate into the nucleus (317). Nuclear YAP and TAZ drive the expression of genes involved in tumor cell proliferation, survival, angiogenesis, EMT, stemness, and drug resistance.

### 6.5 Activation of the Adenylyl Cyclase-cAMP-PKA pathway

GPER-mediated transcriptional regulation occurs indirectly through the cAMP and EGFR signaling pathways. Upon activation by E2, GPER signals via heterotrimeric G-protein, where the Gα*_s_* subunit undergoes activation and stimulates adenylyl cyclase to convert ATP into cAMP, thereby increasing intracellular cAMP levels (318). cAMP acts as a secondary messenger to activate PKA, which phosphorylates transcription factors such as CREB (cAMP response element-binding protein). Phosphorylated CREB then shuttles into the nucleus to induce the expression of genes involved in breast cancer cell proliferation, survival, metabolism, differentiation, metastasis, and therapeutic resistance (refer to **Figure 3C**) (316). In parallel, the Gβγ dimer activates SRC tyrosine kinase, which subsequently activates integrin α5β1 and matrix metalloproteinase (MMPs), leading to EGFR trans-activation (297, 316). These interconnected signaling events highlight the multifaceted role of GPER in driving BC progression.

### 6.6 GPER & EGFR trans-activation, activation of MAPK/ERK pathway

EGFR plays a pivotal role in GPER-mediated signaling in BC (319), particularly contributing to survival, proliferation, migration, and metastasis in ER-positive tamoxifen-resistant tumors. Upon GPER activation, MMPs cleave pro-heparin-binding epidermal growth factor (pro-HB-EGF), releasing HB-EGF, which binds to and activate EGFR (**Figure 3C**). This EGFR transactivation initiates downstream signaling pathways, including MAPK/ERK1/2 and PI3K/Akt, promoting breast cancer cell survival and proliferation (320). Moreover, EGFR ligands have been shown to upregulate GPER expression through the EGFR/ERK pathway, further reinforcing tamoxifen resistance in ER-positive BC. Hypoxic conditions within the tumor microenvironment also induce GPER upregulation via HIF-1α in an EGFR/ERK dependent manner (321). These findings highlight the interconnected nature of EGFR and GPER signaling in BC progression and therapy resistance. Consequently, dual-targeting strategies combining EGFR inhibitors (e.g., gefitinib or erlotinib) with GPER antagonists may offer a more effective approach for reducing tumor burden and overcoming tamoxifen resistance in ERα−positive BC.

### 6.7 GPER signaling in breast CAFs

Cancer-associated fibroblasts (CAFs), also referred to as myofibroblasts, constitute the most abundant stromal cell population within the breast tumor microenvironment (TME)—a dynamic and heterogeneous ecosystem comprising immune cells, blood vessels, extracellular matrix (ECM), and stromal elements that surround and interact with tumor cells. CAFs play a critical role in shaping the TME by orchestrating heterotypic cellular interactions and continuously secreting cytokines, chemokines, metabolites, and ECM-remodeling proteins. This contributes to an immunosuppressive or “immune-excluded” phenotype that facilitates tumor progression and promotes tumor immune escape.

CAFs secrete a diverse profile of cytokines (e.g., IL-6, TGF-β) and chemokines (CXCL1, CXCL12, CCL2, CCL5), which preferentially recruit immunosuppressive cell subsets such as myeloid-derived suppressor cells (MDSCs) and CD4^+^CD25^+^Foxp3^+^ regulatory T (Treg) cells, while inhibiting the cytotoxic activity of CD8^+^ T cells and natural killer (NK) cells. In addition, CAFs actively polarize tumor-associated macrophages (TAMs) and neutrophils (TANs) toward protumor phenotype (M2 and N2, respectively) via factors like IL-4, IL-6, IL-8, GM-CSF, CXCL8, and CXCL12 (322, 323).

GPER is highly expressed in CAFs and functions as a transcriptional regulator in response to estrogen or the GPER agonist G-1. Upon activation, GPER stimulates the paracrine secretion of chemotactic, angiogenic, and ECM-modulating factors, including IL-6, IL-8, VEGF, HGF, and matrix metalloproteinases (MMP-2, MMP-9) (324, 325), which collectively enhance processes such as F-actin reorganization, EMT, migration, and angiogenesis (326–328).

Under hypoxic conditions—commonly observed within tumors—CAFs upregulate HIF-1α, GPER, and α-SMA, leading to increased secretion of IL-6, VEGF, and connective tissue growth factor (CTGF). GPER activation promotes invasion through a CTGF-dependent mechanism, while silencing GPER in CAFs downregulates hypoxia-induced CTGF expression and suppresses BC invasion (329). Estrogen and G-1 have also been shown to elevate HIF-1α and VEGF levels, further promoting tumor angiogenesis (326, 330, 331).

Moreover, Pupo et al. demonstrated that estrogen induces nuclear translocation of GPER in CAFs, upregulating c-Fos and CTGF expression and enhancing fibroblast migration (332). Ligand-activated (E2 and G-1) GPER can also trigger a feedforward loop in both CAFs and MCF-7 cells through IL-1β/IL1R1 signaling, reinforcing invasive characteristics in breast cancer cells (333). Notably, GPER mediates tamoxifen-induced aromatase expression in both CAFs and tamoxifen-resistant BC cells, increasing local estrogen synthesis and driving resistance mechanisms (275, 324). Furthermore, CAF-derived CXCL12 facilitates tumor cell intravasation and metastasis by increasing vascular permeability and promoting leaky tumor vasculature (334). IL-6 from CAFs also promotes cancer stemness by inducing the formation of BCSCSs, which exhibit self-renewal capacity and therapy resistance.

Together, these findings highlight GPER’s central role in CAF biology, particularly in fostering a supportive TME that drives breast cancer progression. Targeting GPER in CAFs represents a promising therapeutic strategy to disrupt stromal support, attenuate immune evasion, and inhibit tumor advancement in ERα−positive BC. The use of GPER antagonists may be especially beneficial as an adjuvant therapy in ERα−positive breast cancer by enhancing immune infiltration and reducing tumor proliferation.

### 6.8 Controversies on GPER

Controversy remains regarding GPER’s role in pro-apoptotic signaling and its subcellular localization. While GPER is classified as a cell-surface transmembrane receptor, studies have reported its presence both at the plasma membrane and intra-cellularly, with distinct biological implications across BC subtypes. Thomas et al. and Filardo et al. observed that GPER primarily exhibits a cytoplasmic staining pattern in BC cells, with a minor fraction at the cell surface (277, 335). However, tumor specimens often show both nuclear and cytoplasmic GPER localization. Cheng et al. demonstrated that GPER accumulates in the perinuclear region and distributes in the cytoplasm via clathrin-coated vesicles (336), raising questions about its role as a membrane-localized estrogen receptor. Sjöström et al. reported that GPER over-expression and plasma membrane localization are key drivers of BC progression, with high membrane GPER correlating with poor histological grade, while its absence predicts excellent long-term prognosis in ER-positive tamoxifen-treated patients (296). In contrast, cytoplasmic GPER is linked to lower tumor stage and better differentiation, whereas nuclear GPER correlates with aggressive subtypes with poorly differentiated tumors (337). GPER’s role in pro-apoptotic signaling remains controversial, with its effects varying depending on the cellular context and signaling environment. Some studies suggest that GPER activation inhibits cancer cell growth (338), implying that high GPER expression may benefit the survival of BC patients, while others report that GPER induces the expression of genes involved in tumor cell migration and proliferation both *in vitro* and *in vivo* (339, 340). Moreover, high GPER expression correlates with increased tumor size and metastasis in breast malignancies (335). Additionally, GPER’s involvement in tamoxifen resistance adds further complexity; while some studies report that high GPER expression is negatively-associated with relapse-free survival in BC patients treated with tamoxifen, others suggest it may enhance treatment sensitivity. Collectively, these findings underscore the need for further investigation to clarify GPER’s dual role as both a pro- and anti-tumorigenic factor and to better understand its functions across diverse pathophysiological contexts, including ERα−positive BC.

## 7 Next-generation therapeutic strategies targeting ERα and GPER

Targeted protein degradation (TPD) has emerged as a promising front-line endocrine therapy, offering specific and irreversible silencing of ER by manipulating cellular proteostasis (341, 342). SERDs induce ERα degradation by binding to the ERα−LBP and recruiting the cellular degradation machinery. The first-generation SERD, fulvestrant (Faslodex™), features a core structure that fits into the ERα−LBP and a hydrophobic alkyl-side chain (degron) that binds to a hydrophobic pocket of ERα. This induces structural deformation of ERα, including the displacement or rearrangement of helix 12, which exposes hidden degradation signals. This facilitates the attachment of ubiquitin molecules to degron sequences, leading to ERα degradation. (343). In this section, we discuss recent advancements in fulvestrant and its analogues, highlighting novel innovations such as ER-targeting PROTACs, CERANs, SERCAs, and other emerging technologies (67, 344, 345).

### 7.1 Fulvestrant and its analogues

Presently, fulvestrant remains the only SERD approved for use in ET-resistant metastatic BC, both as a first-line and subsequent-line treatment (78). However, fulvestrant has several limitations, including low solubility, poor oral bioavailability requiring painful intramuscular administration, a bulky steroidal backbone that restricts chemical diversification, and the emergence of drug resistance due to mutations in ERα−LBP that impair binding and degradation (78, 346–349). These limitations have restricted the full clinical potential of fulvestrant, with ER blockade remaining below 75% even at a monthly dose of 500 mg, thereby spurring the development of second-generation oral SERDs with improved pharmacokinetics (PK) and efficacy (347, 350, 351). Consequently, pharmaceutical efforts have focused on utilizing non-steroidal scaffolds containing two types of chemical moieties—either an acid side chain or basic side chain—that perturb the ERα−LBD and interfere with co-activator binding (352). However, the clinical outcomes of these newly developed oral SERDs have varied so far.

Oral SERDs with acrylic acid side chains undergoing clinical trials include rintodestrant (G1T48), taragarestrant (D-0502), ZN-c5, and LX-039. The early SERD GW5638 was designed based on the tamoxifen core structure by substituting its piperidine side chain with acrylic acid side chain (64, 353). Rintodestrant, developed by G1 therapeutics, demonstrated excellent safety and tolerability in a Phase II clinical trial (NCT03455270) as a monotherapy and in combination with palbociclib in ER+/HER2− advanced BC patients with *ESR1* mutations (354, 355). Similarly, the Phase Ib study of D-0502 (NCT03471663) showed promising anti-tumor activity and tolerable toxicity in patients with ER+/HER2− advanced or metastatic BC (254, 356). D-0502 is currently under evaluation in a Phase III study (CTR20190092). ZN-c5, developed by Zentalis, has demonstrated an excellent safety profile and is being evaluated in a Phase II trial as a monotherapy (NCT03560531) and in Phase I trials in combination with palbociclib (NCT03560531) and abemaciclib (NCT04514159) (257–259). LX-039, an indole-series compound from Luoxin Pharmaceuticals, demonstrated favorable pharmacokinetics and potent anti-tumor activity in wild-type and tamoxifen-resistant MCF-7 xenograft models (255, 357). It is currently in a Phase I trial (NCT04097756) for treating ER+/HER2− advanced or metastatic BC (256).

In contrast, oral SERDs with basic side chains include elacestrant (RAD-1901), imlunestrant (LY3484356), camizestrant (AZD9833), and giredestrant (GDC-9545). Elacestrant, a second-generation SERM-SERD hybrid developed by Stemline Therapeutics, received FDA approval under the brand name Orserdu^®^ in 2023 (358, 359). The Phase III EMERALD trial (NCT03778931) demonstrated that elacestrant, as a single agent, significantly improved PFS compared to standard-of-care (AI or fulvestrant) in patients with ER+/HER2−, *ESR1*-mutated advanced or metastatic breast cancer previously treated with ET and a CDK4/6 inhibitor (57, 58, 360). The ongoing Phase Ib/II ELECTRA trial (NCT05386108) is evaluating elacestrant in combination with abemaciclib for treating brain metastases in ER+/HER2− breast cancer patients (361), suggesting that elacestrant could become a new standard-of-care in this setting.

Camizestrant (AZD9833), developed by AstraZeneca, demonstrated superior efficacy and tumor inhibition in patients with ER+/HER2− advanced breast cancer compared to fulvestrant in the Phase II SERENA-2 trial (NCT04214288) (241, 242, 362–365). The ongoing Phase-III SERENA-6 trial (NCT04964934) is evaluating its antitumor activity as a single agent or in combination with CDK4/6 or PI3K/AKT/mTOR inhibitors in fulvestrant-resistant, wild-type, and *ESR1*-mutated PDX models (59).

Imlunestrant (LY3484356), developed by Loxo Oncology at Eli Lilly Corp., is a next-generation brain-penetrant, oral selective ERα degrader that exhibits potent activity in both *ESR1* wild-type and mutant breast cancers (29, 60). The ongoing Phase I/II EMBER trial (NCT04188548) is assessing the safety and efficacy of imlunestrant as monotherapy and in combination with other anticancer agents in patients with ER+ locally advanced or metastatic breast cancer (248). When combined with abemaciclib (a CDK4/6 inhibitor), alpelisib (a PI3K inhibitor), or everolimus (a mTOR inhibitor), imlunestrant demonstrates enhanced anti-tumor efficacy, including against brain metastases, irrespective of *ESR1*-mutation status (29). According to the ongoing Phase-III EMBER-3 trial (NCT04975308), the imlunestrant–abemaciclib combination significantly improves PFS compared to imlunestrant monotherapy in ER+/HER2− advanced breast cancer, regardless of *ESR1* mutations (61).

Giredestrant (GDC-9545), developed by Genentech, is a highly potent, non-steroidal oral SERD and full ER antagonist. Phase I clinical data indicate that GDC-9545 is well tolerated and demonstrates promising efficacy both as a monotherapy and in combination with palbociclib (366, 367). Notably, at low doses, GDC-9545 induces tumor regression in both wild-type ERα tumor models and Y537S ERα mutant PDX models, either alone or in combination with a CDK4/6 inhibitor (62). Ongoing Phase III trials—persevERA (NCT04546009) and evERA (NCT05306340)—are evaluating its efficacy and safety in combination with palbociclib and everolimus, respectively, in ER+/HER2− locally advanced or metastatic breast cancer patients (245, 247).

However, clinical development of several new SERDs—such as AZD9496 (368–370), LSZ102 (371, 372), GDC-0810 (373–376), GDC-0927 (377, 378), SCO-120 (64, 379), SHR9549 (64) and SAR439859 (380–384)— has been suspended due to various concerns.

### 7.2 ER PROTACs

**Figure 6:**
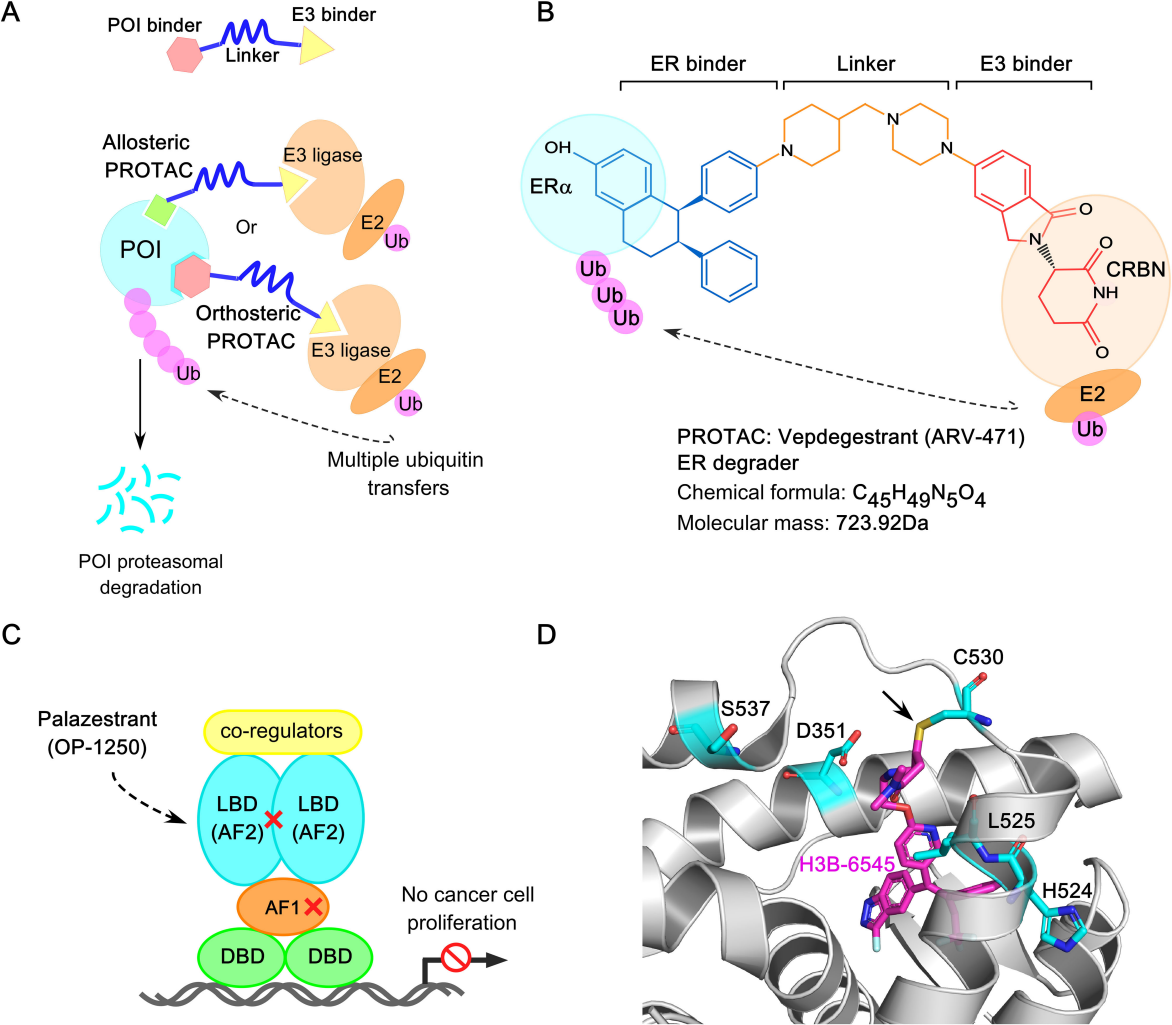
Next-generation protein degradation technologies for ER+ breast cancer therapy. **(A)** Mechanism of action of allosteric and orthosteric PROTACs, leading to proteasomal degradation of protein of interest (POI). **(B)** Chemical structure of oral ER-PROTAC ARV-471. **(C)** Mechanism of action of CERAN OP-1250. It completely turns off both AF1 and AF2 transcriptional activation function of ERα. **(D)** Crystal structure of H3B-6545 (in purple sticks) in complex with ER (PDB: 6OWC), highlighting the co-valent bonding between Cysteine 530 (C530) of ER and H3B-6545 (indicated with arrowhead).

Proteolysis-targeting chimera (PROTAC) technology, first proposed by Sakamoto et al., is an emerging TPD strategy (66, 385–387). PROTACs are heterotrimeric bifunctional molecules consisting of three components: a ligand that binds to the protein of interest (POI), a ligand that binds to an E3 ubiquitin ligase, and a flexible linker connecting them. PROTACs induce the formation of a “POI-PROTAC-E3 ligase” ternary complex and, by “hijacking” the cellular ubiquitin-proteasome system (UPS), trigger POI ubiquitination and subsequent degradation via the proteasome pathway (388). In this context, orthosteric PROTACs target the active-site of the POI, whereas the allosteric PROTACs bind to a site distinct from the primary-ligand-binding pocket (**Figure 6A**). The rational design of small-molecule ER PROTACs—most notably the Von Hippel-Lindau (VHL)-based and Cereblon (CRBN)-based PROTACs—has driven the evolution of the ER PROTAC platform from conceptualization to clinical translation. In VHL-based PROTACs, HIF-1α or other small molecules serve as warheads (ligand-binding moieties) to recruit the VHL E3 ligase, whereas thalidomide and its derivatives act as warheads to engage the CRBN E3 ligase in CRBN-based PROTACs. The warhead for ERα generally includes E2, SERM/SERD, peptide, or DNA fragment. Notably, PROTACs are catalytic in nature, meaning they can be recycled after each degradation event to target additional POI molecules, distinguishing them from stoichiometric degraders. A key advantage of PROTACs over SERDs is that they do not require high-affinity binding to the ligand-binding pocket of the POI, allowing structural modifications to improve solubility without compromising efficacy.

### 7.3 CRBN-based ER PROTAC degraders

The pioneering ER PROTAC ARV-471 (Vepdegestrant), developed by Arvinas and Pfizer, entered clinical trials in 2019 and received FDA fast-track designation in February 2024 (64, 389, 390). ARV-471 is a CRBN-based PROTAC, incorporating a lasoxifene-derived ligand-binding moiety (**Figure 6B**) (65, 391). It simultaneously binds to the ER-LBD and the CRBN E3 ligase, facilitating the degradation of both wild-type and mutant ERα at nanomolar concentrations. Gough et al. reported that ARV-471 selectively and rapidly degraded ER, achieving >80% degradation within 4 hours across various ER+ cell lines, and demonstrating equal potency against clinically relevant ligand-independent ERα mutants (392). The phase III VERITAC-2 trial (NCT05654623) is currently evaluating the efficacy and safety of vepdegestrant versus fulvestrant, while VERITAC-3 (NCT05909397) is assessing vepdegestrant plus palbociclib versus letrozole plus palbociclib in patients with ER+/HER2− advanced breast cancer (266, 268, 269, 393). Additionally, ARV-471 is being explored in combination therapies with agents such as abemaciclib, ribociclib, everolimus, and Pfizer’s novel CDK-4 inhibitor (PF-07220060), expanding its potential applications for locally advanced or ER+/HER- metastatic BC (NCT06125522, NCT05573555, NCT0558127) (64).

ERD-3111 (compound 18) was reported as a novel CRBN-based ER PROTAC by the Wang group in 2023 (63, 64). This chimera utilized lasoxifene as the ER binder and incorporated a new CRBN ligand, TX-16. Notably, ERD-3111 demonstrated superior bioavailability and achieved significant tumor regression and complete growth inhibition in wild-type and two clinically relevant *ESR1*-mutated (Y537S and D538G) MCF-7 xenograft models, outperforming ARV-471. Based on these preclinical findings, ERD-3111 is being extensively evaluated as a highly potent ERα PROTAC for further development. Subsequently, the development of more potent and orally efficacious CRBN-based ER PROTACs led to ERD-1233 (compound 19) and ERD-12310A (compound 20), which utilize the lasoxifene scaffold as the ER-binding moiety and a novel CRBN ligand with high binding affinity (394, 395). Importantly, ERD-12310A exhibited significant inhibition of tumor growth in MCF-7 Y537S ERα mutant xenograft tumors without substantial weight loss or toxicity issues, making it more effective than ARV-471.

### 7.4 VHL-based ER PROTAC degraders

Besides CRBN ligands, the VHL ligand is also widely employed as an E3 ligase recruiter for designing ER PROTACs (396). A novel VHL-based ER PROTAC, AC0682, was developed by Accutar Biotech using an AI-empowered drug discovery platform with ACCU degron technology. Although AC0682 was reported to induce ERα degradation in wild-type and Y537S/D538G ERα−expressing MCF-7 cell lines with a sub-nanomolar DC_50_, its Phase I clinical trials (NCT05489679 and NCT05080842) were recently terminated. The next-generation AC699 is currently recruiting patients to evaluate its safety, tolerability, PK, and efficacy in ER+/HER2− advanced or metastatic BC, though its chemical structure remains undisclosed.

Other highly potent VHL-based ER PROTAC degraders, ERD-308 (compound 12) and ERD-148 (compound 11), were developed by the Wang group at the University of Michigan in 2019 (396–398). These compounds employed a raloxifene scaffold as the ER ligand and exhibited excellent ER-degrading potency. Notably, ERD-148 degrades both unphosphorylated and phosphorylated ERα, resulting in greater suppression of E2-dependent wild-type and E2-independent *ESR1*-mutated (Y537S and D538G) MCF-7 cells.

In 2022, another innovative class of ER PROTACs targeting the DBD of ERα—termed ERE-PROTACs (a nucleic acid conjugate)—was developed by the Tan group from Tsinghua university to overcome ET resistance (399). In a subsequent study, Feng et al. proposed an aptamer PROTAC strategy for targeting ERα−DBD to overcome drug resistance, using the aptamer as a ligand for ERα and the small-molecule VH032 for recruiting VHL E3 ligase (389). In 2023, another novel class of dual-targeting PROTAC degraders designed to simultaneously degrade ERα and aromatase (ARO) was introduced. Among these, 18c (compound 16) exhibited the most potent dual ERα/ARO degradation activity (400).

### 7.5 Complete estrogen receptor antagonists (CERANs)

OP-1250 (Palazestrant), developed by Olema, is the only orally bioavailable CERAN in clinical trials, effectively targeting both wild-type and mutant ERα (401). Unlike SERMs, CERANs are designed to completely suppress AF1 and AF2 activity, while also functioning as SERDs to promote ER degradation (**Figure 6C**). The Phase III OPERA-1 trial (NCT06016738) is currently evaluating the safety and efficacy of OP-1250 versus standard-of-care in patients with ER+/HER2− advanced breast cancer (265). Combination therapies of OP-1250 with palbociclib, ribociclib, and alpelisib are also being assessed in Phase I/II trials (NCT05266105, NCT05508906) (402).

### 7.6 Selective estrogen receptor covalent antagonists (SERCAs)

H3B-6545, a first-in-class oral SERCA, was discovered using a structure-based drug design strategy. It irreversibly inactivates both wild-type and mutant ERα through co-valent bond formation between the cysteine 530 (C530) in the ERα−LBD and the acrylamide warhead of H3B-6545 (PDB: 6OWC) (**Figure 6D**) (68). H3B-6545 demonstrates robust preclinical anti-tumor efficacy and superiority over fulvestrant across a wild-type and Y537S-mutant ERα−expressing models, including both palbociclib-sensitive and -resistant BC lines. Its clinical activity is being evaluated in ER+/HER2− metastatic BC, including patients harboring Y537S ERα, in trials NCT03250676, NCT04568902, and NCT04288089 (68, 260, 262, 403). While H3B-6545 enforces an antagonistic conformation without degrading ERα, compound 29c targets C530 covalently and engages in strong hydrophobic interactions with helix 11, demonstrating ERα degradation potency in both wild-type and *ESR1*-mutated BC cell lines (404).

### 7.7 Limitations of PROTACs

Despite the groundbreaking success of PROTAC technology, several technical challenges remain, including expanding the repertoire of E3 ligases, reducing off-target toxicity, and optimizing linker length—all of which hinder further development. The limited availability of E3 ligases further restricts its application. Similarly, other UPS-based modalities, such as autophagy-targeting chimeras (AUTACs), autophagosome-tethering compounds (ATTECs), molecular glues, dTAG, SNIPERs, and Trim-Away, face similar constraints (344, 405–409).

### 7.8 GPER-targeting strategies

Analysis of breast cancer biopsy samples based on ER and GPER expression reveals that 43% of cases are ER+/GPER+, 19% are either ER+/GPER- or ER-/GPER+, and 19% are ER-/GPER- (**Figure 3B**) (410). This suggests that standard ER-targeted therapies fully benefit only 19% of patients, partially benefit 43%, and overlook a substantial proportion of GPER-expressing tumors—highlighting a critical gap in current endocrine therapy.

Encouragingly, GPER-directed therapeutic strategies are emerging. For instance, the GPER agonist LNS8801 significantly inhibited tumor growth in uveal melanoma xenografts by inducing G2-M phase mitotic arrest and apoptosis (411). A Phase 1/1B clinical trial (NCT04130516) is currently evaluating LNS8801 as monotherapy and in combination with pembrolizumab in metastatic solid tumors, with early results demonstrating promising safety and efficacy.

In parallel, two dual ER/GPER-targeting PROTACs, UI-EP001 and UI-EP002, have shown nanomolar binding affinities and effectively degrade ERα, ERβ, and GPER (412). However, the broader application of such strategies remains limited by the scarcity of selective GPER modulators—both agonists and antagonists—constraining efforts to fully characterize GPER-mediated signaling in breast cancer.

While PROTACs have revolutionized intracellular protein degradation by harnessing UPS, they are generally ineffective against non-cytosolic and membrane-associated targets like GPER. To address this, novel degradation technologies such as antibody-based PROTACs (AbTACs) and lysosome-targeting chimeras (LYTACs) have gained traction. These approaches enable the selective degradation of transmembrane and extracellular proteins by directing them to the lysosomal pathway, potentially expanding the therapeutic options for previously “undruggable” targets. In the context of GPER, AbTAC and LYTAC strategies offer a promising avenue for overcoming the limitations of traditional degraders and hold significant clinical potential for ER+/GPER+ breast cancer (390, 413).

**Figure 7:**
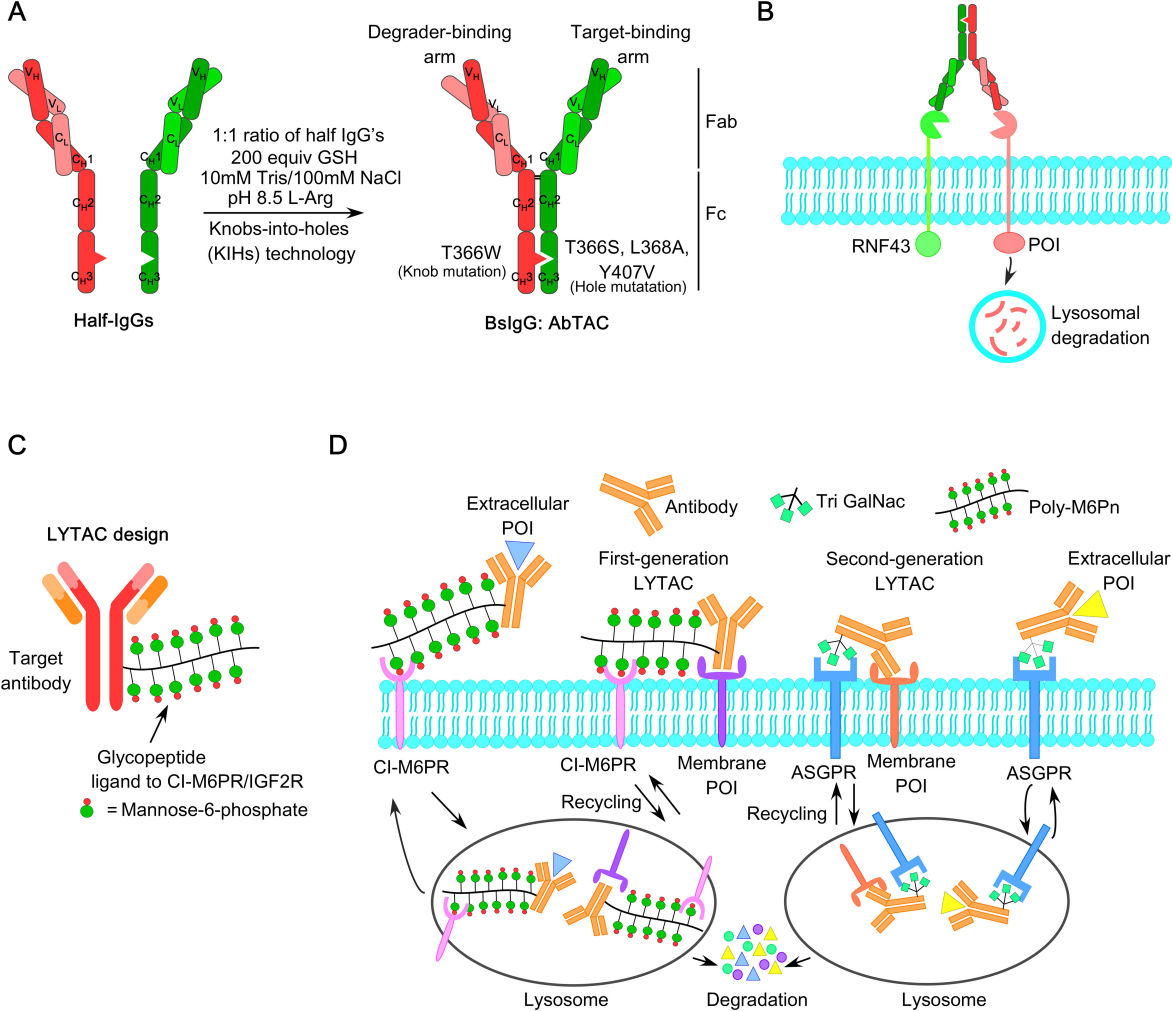
AbTAC- and LYTAC-based degradation strategies for targeting membrane-bound receptors **(A)** Generation of an AbTAC bispecific IgG that simultaneously binds to RNF43 and PD-L1, modified from Cotton et al. (414). The conditions for *in vitro* assembly of individually expressed half-IgGs to form a bispecific IgG with the desired point mutations are described. Using Knobs-into-holes (KIHs) Fc engineering, one half-IgG contains the T366W ‘knob’ mutation, substituting threonine with bulkier tryptophan, while the other half-IgG contains the T366S, L368A, Y407V mutations with a complementary ‘hole’. **(B)** Graphical representation of the AbTAC mode of action, recruiting RNF43 for lysosomal degradation of membrane-bound POI. **(C)** Structure of LYTAC utilizing glycopeptide ligand to target CI-M6PR/IGF2R. **(D)** Mechanism of action of first-generation and second-generation LYTACs for degrading extracellular POI or membrane-bound POI, recycling CI-M6PR and ASGPR receptors respectively.

AbTAC are bispecific IgGs that simultaneously bind two distinct proteins (**Figures 7A, B**). Cotton et al. developed the first AbTAC, which targets RNF-43 (an E3 ligase) and programmed death-ligand 1 (PD-L1), promoting PD-L1 lysosomal degradation (414). Using Knobs-into-Holes (KIH) Fc engineering, one half-IgG incorporates the T366W ‘knob’ mutation—substituting threonine with the bulkier tryptophan—while the other half-IgG carries the T366S, L368A, and Y407V mutations to form the complementary ‘hole’. In addition, the N297G mutation is introduced to prevent Fc glycosylation, thereby silencing the Fc region and reducing antibody flexibility during AbTAC generation.

In contrast, LYTACs, developed by the Bertozzi lab, consist of a small-molecule or antibody fused with a glycopeptide ligand recognized by cation-independent mannose-6-phosphate receptors (CI-M6PR), which shuttle M6P-tagged protein cargoes to lysosomes for degradation while recycling themselves (**Figures 7C, D**) (415, 416). Atezolizumab-derived LYTACs (anti-PD-L1-M6Pn) achieved ∼70% PD-L1 degradation via M6P recognition, while ASGPR-targeting LYTACs demonstrated liver-specific EGFR degradation (415, 417). Rational design of GPER-targeted warheads for LYTACs or AbTAC-drug conjugates (ATDCs) holds promise for degrading membrane-bound GPER, blocking downstream signaling, and enabling intracellular delivery of conjugated drugs in the treatment of ER+/HER2− advanced BC patients.

## 8 Conclusion

The reliance on ER signaling in ERα−positive breast cancer underscores the importance of ER-targeted therapies as the cornerstone of treatment for this tumor type. The high prevalence of *ESR1* point mutations in ERα−positive metastatic tumors indicates that ER dependency persists throughout tumor progression, driving acquired resistance (418). Functional and structural studies have demonstrated that common mutations such as Y537S and D538G stabilize ERα in a conformation resembling the ligand-bound wild-type receptor, leading to constitutive, hormone-independent activity and resistance to conventional endocrine therapies (51, 52, 207, 210, 419). Crystallographic and modeling analyses reveal that helix 12 in the mutant receptor adopts an “on-state” conformation similar to the E2-bound wild-type ERα, emphasizing the need for novel therapeutics capable of overcoming this constitutively active state while preserving structural integrity to ensure inactivity in the absence of estrogen.

Substantial efforts thus have been directed toward the development of new-generation of ER-targeted agents, including oral SERDs and innovative strategies such as PROTACs, SERCAs, and CERANs. While next-generation SERDs and SERM/SERD hybrids have demonstrated efficacy in targeting ERs, their dependence on ligand binding and potential GPER agonism necessitate more comprehensive approaches. Rigorous evaluation of these agents is ongoing, with multiple preclinical and clinical trials underway in both primary and metastatic breast cancer. Currently, several candidates are in Phase III clinical trials, including camizestrant (AZD9833, AstraZeneca), Taragarestrant (D-0502, Inventis Bio), giredestrant (GDC-9545, Roche), Imlunestrant (LY3484356, Eli Lilly), and palazestrant (OP-1250, Olema Pharmaceuticals), either as monotherapy or in combination with CDK4/6 inhibitors, PI3K inhibitors, and mTOR inhibitors.

On contrary, targeted protein degradation (TPD) has emerged as a transformative strategy for addressing “undruggable” protein targets, with PROTAC technology revolutionizing traditional therapeutic paradigms. ARV-471 has demonstrated exceptional efficacy in Phase I/II trials and is currently in Phase III, positioning it as the first oral ER-targeting PROTAC with strong clinical potential (420). Nanoengineered-PROTACs (nano-PROTACs), such as ARV-loaded nanoparticles, have improved drug solubility, permeability, pharmacokinetics, and intracellular delivery—enhancing efficacy while minimizing systemic toxicity (421, 422). Additionally, surface modification of PLGA nanoparticles with PEG conferred high serum stability and extended half-life to c-Myc-targeting PROTACs in pancreatic cancer models (423, 424). Trastuzumab-conjugated PROTAC-loaded nanoparticles (MZ1-loaded polymeric antibody-conjugated nanoparticles) have also demonstrated enhanced specificity and cytotoxicity in HER-2 enriched BC (425).

Despite the advances, challenges remain in this evolving field, including optimization of linker length, ternary complex equilibria, pharmacokinetics, and the possibility of potential drug resistance of PROTACs. Emerging strategies such as AbTACs and LYTACs, supported by AI-driven platforms for high-throughput screening and rational designing, may represent the next frontier (426). Notably, the development of AbTAC-drug conjugates (ATDCs) targeting membrane proteins like GPER offers a dual benefit: receptor degradation and intracellular drug delivery. This approach addresses previously inaccessible targets and paves the way for more effective treatment options in ER+/HER2− breast cancer. Overall, continuous breakthroughs and refinements in PROTAC technology and related TPD strategies offer promise for developing safer, more precise, and controllable ER-targeting therapeutics—potentially transforming the treatment landscape for ERα−positive breast cancer patients.
